# Applications of Microextraction Technology for the Analysis of Alcoholic Beverages Quality: Current Perspectives and Future Directions

**DOI:** 10.3390/foods14071152

**Published:** 2025-03-26

**Authors:** Yue Qiu, Qi Deng, Yongqing Zhang, Baoguo Sun, Wenxian Li, Wei Dong, Xiaotao Sun

**Affiliations:** 1 Beijing Laboratory of Food Quality and Safety, Beijing Technology and Business University, Beijing 100048, China; qiuyue010122@163.com (Y.Q.); 15378183088@163.com (Q.D.); yongqing2023@163.com (Y.Z.); sunbg@btbu.edu.cn (B.S.); liwenxian0502@163.com (W.L.); 2 Key Laboratory of Brewing Molecular Engineering of China Light Industry, Beijing Technology and Business University, Beijing 100048, China; 3 Key Laboratory of Geriatric Nutrition and Health Ministry of Education, Beijing Technology and Business University, Beijing 100048, China

**Keywords:** microextraction, quality, alcoholic beverages, liquid-phase microextraction, solid-phase microextraction

## Abstract

Alcoholic beverages are loved by the majority of consumers because of their diverse characteristics and rich nutritional value; thus, ensuring their quality is necessary for maintaining the rapid development of the alcoholic beverage industry. Due to trace levels of various quality factors and the complexity of the beverage body matrix, pretreatment is usually required before analysis. Among the many pretreatment methods available, microextraction has attracted increasing attention because it aligns with the development direction of green chemistry. This review surveys advancements in microextraction techniques pertaining to three quality aspects in the most frequently consumed alcoholic beverages: baijiu and huangjiu (spirits) and wine and beer (fermented alcoholic drinks). Furthermore, new directions in their development are discussed.

## 1. Introduction

Alcoholic beverages (ABs) are produced and consumed worldwide. It can be roughly divided into three categories according to different production processes or the content of alcohol: distilled spirits (baijiu, vodka, brandy, etc.), fermented alcoholic beverages (wine, beer, etc.), and integrated alcoholic beverages. Currently, over 2000 compounds have been identified in baijiu, while more than 1000 and 3000 distinct compounds have been detected in wine and beer [1]. These substances constitute the unique sensory experience and rich nutritional properties of ABs. For example, beer is rich in soluble fiber, minerals, and vitamins [2], which have a preventive effect on cancer, vascular diseases, and digestive system diseases. Due to these characteristics, alcoholic beverages are deeply loved and widely consumed by the public. According to Statista, the global alcoholic beverage market revenue in 2024 was approximately USD 1715.95 billion, with a global volume of about 284,288.86 million L [3]. In recent decades, the enormous market value of ABs has prompted numerous enterprises to engage in production, but this also brings about a series of quality issues that require urgent resolution, including flavor deterioration, textural haziness, physiochemical changes during processing or storage, packaging material issues, hazardous compound production, and harmful substance residues. Therefore, ensuring the quality of ABs is not a mere regulatory requirement—it is a critical factor in safeguarding consumer health, safety, and trust and supporting the sustainable development of the entire industry.

The quality of ABs is affected by intrinsic factors (e.g., flavor, functional value, and safety) [4], which are critically related to a complex combination of volatile and non-volatile components, including their interactions. A total of 130 odorants with an odor activity value (OAV) greater than 1, 139 functional substances, and 182 harmful substances found in baijiu, huangjiu, wine, and beer are summarized in Table S1. These substances are key to the quality of ABs; e.g., esters usually exhibit fruity and floral aromas, which contribute to the aromatic quality of ABs, and polyols in ABs have a certain sweetness, which can give roundness to the body of a beverage and add complexity to its mouthfeel. However, some substances can pose health risks and reduce AB safety to unacceptable levels. For example, the methanol present in ABs is a problem; it is toxic to humans, causing severe poisoning when a volume of 5–10 mL is ingested, blindness when more than 10 mL is ingested, and death when 30 mL is consumed [5]. As demonstrated by numerous studies, the types of raw materials, contact materials, and manufacturing processes can influence the formation and concentration of the substances mentioned above. For instance, Xu [6] et al. found that in fortified Daqu inoculated with *Bacillus subtilis* and *Staphylococcus epidermidis*, the abundance of *Pantoea*, *Weissella*, *Staphylococcus*, and *Thermoascus* was relatively high, leading to an increase in the contents of esters and aromatic compounds. Zhao [7] et al. studied the brewing characteristics of glutinous sorghum and non-glutinous sorghum and found that the contents of ethyl acetate, ethyl caproate, and ethyl butyrate in liquor brewed from non-glutinous sorghum were all higher than those in liquor brewed from glutinous sorghum. Through the analysis of the physicochemical properties and starch properties of sorghum, it was found that the total starch and tannin content of non-glutinous sorghum was the highest, which might be the direct cause of the high content of the above substances. Moreover, NTULI R G et al. [8] found that wines fermented at higher temperatures have a more prominent red color, and lower fermentation temperatures increase the concentration of esters. Therefore, only when we have deciphered the changes in the content of the relevant substances in each production process can we know the parameters that need to be controlled, and this requires a comprehensive analysis of the composition of ABs. ABs have a complex composition involving a wide variety of substances, some of which are present at extremely low levels and are easily altered; e.g., sulfur compounds are usually present in ABs at low levels and low thresholds, contributing to the odors of ABs, i.e., savory and oniony odors, making it challenging to comprehensively and accurately analyze their composition, which requires effective analytical methods. The analysis of ABs often encompasses both subjective and objective dimensions. The subjective analysis primarily involves consumers or professional evaluators who assess the aroma, taste, color, and other sensory attributes of ABs through their senses. Sensory analysis typically requires specialized training; however, sensory results are still susceptible to individual states and external environmental influences. Objective analysis, on the other hand, utilizes various instruments to analyze the components of ABs. Compared to subjective analysis, instrumental analysis is more accurate, reproducible, objective, and unaffected by fatigue or other similar factors [9]. Recently, AB quality analyses involved the widespread use of methods such as chromatography, spectroscopy, and separation techniques that can be combined with mass spectrometry (such as LC-MS and GC-MS) to enable the high-throughput and high-sensitivity analysis of components [10]. Moreover, testing technologies such as NMR and FT-IR can achieve rapid and non-destructive detection. However, neither of these techniques can effectively distinguish between complex matrices and detect low-concentration substances [11]. Therefore, pretreatment techniques are necessary to achieve reliable analysis. At present, several methods have been commonly used for AB pretreatment, such as direct injection (DI), liquid-liquid extraction (LLE), liquid-liquid microextraction (LLME), solid-phase extraction (SPE), solid-phase microextraction (SPME), stir bar sorptive extraction (SBSE), solvent-assisted flavor evaporation (SAFE), simultaneous distillation extraction (SDE), and purge and trap (P&T) [12]. Among the various extraction methods, traditional extraction technologies such as LLE and SPE can achieve the efficient detection of AB targets. However, these traditional methods also exhibit problems, such as high solvent and sample consumption and high labor requirements, and are time-consuming. Hence, there is a trend towards reducing organic solvent use for sample preparation or reducing sample use [13]. Furthermore, with the introduction of “green chemistry”, an increasing number of researchers have improved traditional methods and derived new extraction methods, such as microextraction; microwave-assisted, ultrasonic-assisted, and membrane-assisted extraction; and supercritical fluid extraction [14]. Among these, microextraction techniques are a particularly promising development, offering significant advantages in terms of reducing solvent usage, sample volume, and environmental impact.

Microextraction techniques refer to extraction methods that use little or no solvent or miniaturize simple extraction methods. Techniques such as SPME and liquid-phase microextraction (LPME) are widely used in food analysis. These methods are applied to analyze and determine various contaminants in food, including agricultural pollutants, environmental toxins, processing-induced pollutants, food contact materials, illegal additives, and other undesirable substances. Additionally, microextraction techniques are also valuable for analyzing the sensory qualities of food, such as volatile aromatic compounds [15]. To date, these techniques have been widely applied in the preparation of AB samples. However, not all microextraction techniques are perfectly suited for the analysis of the various quality factors in ABs. For example, Dispersive liquid-liquid microextraction (DLLME) is widely used in the analysis of contaminants in ABs, while flavor analysis is less common. We researched the application of DLLME in ABs and found that the ratio of the application in contaminant analysis to that in aroma analysis was 5:2. In addition, some problems pertaining to microextraction methods commonly used in the analysis of ABs persist and need to be solved, e.g., the matrix effect and the weak enrichment ability of SPME. Therefore, to fully understand the quality attributes of ABs and their influencing factors, it is crucial to understand the current status of the application of microextraction techniques in the analysis of AB quality, existing problems, and future development trends for the quality control of ABs and for microextraction techniques used to analyze ABs.

In this review, we comprehensively summarize the types of substances related to AB quality, their contribution to flavor, and their functional and harmful characteristics. This study also systematically reviews the principles, development status, advantages, and disadvantages of microextraction techniques applied for the analysis of AB quality and discusses existing problems in their development, their future development directions, and their potential application prospects. These summaries will promote an in-depth understanding of the quality of ABs and microextraction techniques.

## 2. Substances Related to AB Quality and Their Key Influencing Factors

### 2.1. Substances That Affect AB Quality

At present, the quality of ABs is mostly determined by intrinsic factors such as flavor substances (appearance, aroma, and taste), functional substances, and harmful substances [4], a summary of which can be found in Table S1. The levels of these substances determine the quality of an AB.

#### 2.1.1. Flavor Substances

An AB’s flavor is closely related to its chemical components, among which volatile components are the primary influencers, including alcohols, aldehydes, esters, acids, and others. Moreover, non-volatile components also significantly contribute to the AB flavor, including saccharides, phenols, amino acids, and proteins. The contribution of aroma compounds in ABs is often studied using techniques such as aroma extract dilution analysis (AEDA), OAVs, aroma reconstitution, and omission experiments. Compounds with an OAV greater than 1 are considered to be key flavor compounds. According to research by Lin [1] et al., there are 294 key flavor compounds across various types of ABs, including baijiu, huangjiu, rum, whisky, vodka, brandy, beer, and wine. Esters are the most abundant among these compounds; thus, they play a pivotal role in aroma contribution. In the baijiu industry, grades such as special, premium, and first-class are typically determined by the total ester content and the content of some typical esters; e.g., the typical flavor of strong-aroma baijiu is a complex aroma dominated by ethyl hexanoate, which has a fruity scent [16]. When the total ester content is greater than or equal to 2.0 g/L, the baijiu is classified as premium quality, and when the content is greater than or equal to 1.5 g/L, it is classified as first-class quality [17]. However, it is not true that more aroma substances result in a better AB; for example, diacetyl is the main raw green flavor substance in beer, and a diacetyl content of 0.1~0.15 mg/L provides a pleasant aroma to beer, but over 0.15 mg/L, it produces an unpleasant rancid rice flavor, which affects the beer’s organoleptic quality [18]. In addition to the content of individual substances affecting quality, interactions between substances also have an impact on AB aroma quality; for example, when substances such as ethyl lactate, ethyl acetate, and ethyl butyrate are in an appropriate proportion, they contribute to a “cellar aroma”, which is one of the most distinctive features of strong-aroma baijiu and sets it apart from other types of baijiu [19]. In addition to aroma compounds, colored and turbid substances are also important factors that affect organoleptic quality. For example, turbidity in light-colored beer is mainly caused by complexes produced by proteins and polyphenols; a turbidity (EBC) of less than 0.9 is considered superior, and a turbidity of less than 1.2 is considered first-class [20]. In the Chinese national standard, the color of baijiu is required to be clear and transparent, without precipitates, but the solubility of some esters and alcohols present in it, such as ethyl palmitate, ethyl oleate, ethyl linoleate, and other high-level fatty acid esters, decreases with temperature. When the temperature changes in winter, low-temperature precipitation makes baijiu turbid [21], and this deteriorates its organoleptic quality.

#### 2.1.2. Functional Substances

Along with providing a sensory experience, moderate drinking can be beneficial for health. The beneficial effects of moderate drinking on health largely depend on the bioactive components in ABs. Wine is rich in a variety of bioactive substances, such as polyphenols, tannins, anthocyanosides, and resveratrol, which have been proven to be beneficial to human health [22]. Polyphenols are recognized as key factors for antioxidant and anti-inflammatory effects [23]. Studies have found that the proanthocyanidins in grape seeds can quickly inhibit the expression of miR-33 and miR-122 in rat liver cells, thereby reducing the mRNA levels of fatty acid synthase, decreasing fat production, and achieving blood lipid-lowering effects [24]. Resveratrol is a natural antioxidant with anti-aging and lipid-regulating properties [25]. Cao [26] et al. explored the effects of resveratrol on blood lipids in a randomized, controlled trial and found that resveratrol could effectively reduce the levels of total cholesterol, triglycerides, and low-density lipoprotein cholesterol in the blood. Beer is rich in proteins, amino acids, branched-chain oligosaccharides, minerals, vitamins, and other nutrients [27]. Beer contains antioxidants, which are primarily represented by phenolic compounds and melanoidins [28]. Studies have shown that beer polyphenols reduce the risk of cardiovascular diseases by increasing the concentration of nitric oxide in the plasma [29]. Of the thousands of chemical components present in baijiu, 202 compounds have been reported as functional compounds, including phenols, organic acids, esters, aldehydes, pyrazines, sulfur, terpenes, furans, and other volatile compounds, as well as amino acids, biologically active peptides, polyols, vitamins, minerals, and other non-volatile compounds [30]. These substances have multiple functions; for example, 2,3,5,6-tetramethylpyrazine is a common functional factor present in jiang-aroma baijiu and sesame-aroma baijiu, which exhibits various effects such as increasing blood circulation, preventing blood stasis, and treating cardiovascular and cerebrovascular diseases [1]. The presence of these functional components enhances the functional qualities of ABs.

#### 2.1.3. Harmful Substances

ABs act as a double-edged sword, as moderate drinking can be beneficial while excessive drinking can be harmful to health. This is because ABs contain some substances that harm health, such as residual agricultural pollutants, environmental pollutants, natural toxins, process-induced contaminants, food contact materials, illegal additives, and other harmful substances [31]. Pesticide contamination is a problem affecting many types of ABs. Grapes, the raw material used to make wine, are highly susceptible to pests and diseases, and the irrational use of pesticides can lead to pesticide residues or even surfeits in grapes. The maximum residue limit in the EU for carbendazim in wine is 0.5 mg/kg, and for methomyl, it is 1 mg/kg. Although the processing methods in the brewing process can significantly reduce the amount of pesticide residues, such as cold soaking, clarification filtration, and fermentation processes can significantly reduce the pesticide residue content in raw grape juice [32,33], several studies have shown that these residues are widely present in commercially available wine [34]. Acute toxicity may result if residues are high, and chronic toxicity may result from the long-term consumption of wine with low doses of pesticide residues. In addition to residues of exogenous substances, substances produced during the production of ABs may also increase the health hazards associated with their consumption; for example, ethyl carbamate is a carcinogenic substance widely found in ABs and fermented food. It has been classified as a Group 2A carcinogen by the International Agency for Research on Cancer (IARC). Its allowable limit in distilled spirits (e.g., tequila, whisky, and vodka) in the United States is 125 μg/L, and Canada has set its allowable limit in table wine in the range of 15 to 30 μg/L [35]. According to survey results, the detection rate of ethyl carbamate in wine is nearly 100% [36]. Biogenic amines are a class of amino-alkaline organic compounds widely found in fermented ABs such as wine, beer, and huangjiu. Excessive AB intake can cause symptoms of intoxication such as dizziness, nausea, respiratory disorders, and palpitations, and in severe cases, may lead to cerebral hemorrhage or even death [37]. In addition to the aforementioned substances, esters, aldehydes, higher alcohols, methanol, furfural, hydrogen cyanide, phthalate plasticizers, aflatoxins, hesperidin, and heavy metals are also important quality parameters affecting the safety of ABs [38].

### 2.2. Key Factors Affecting the Formation of Substances Related to AB Quality

**Figure 1:**
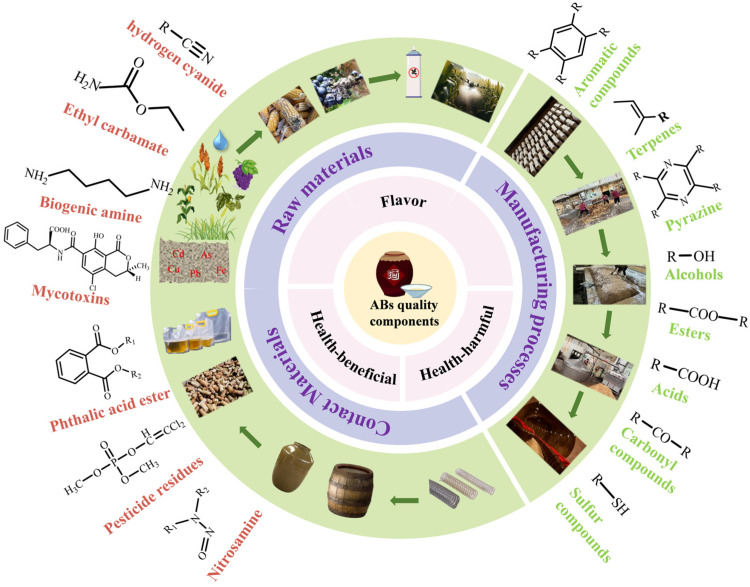
Types of quality factors of alcoholic beverages and their influencing factors. (The outermost layer is the structure of quality-related substances, the green and purple layers are the influences of quality factors, and the pink layer is the types of quality factors).

The substances affecting AB quality can be divided into two categories based on their formation mechanisms, which can be seen in Figure 1: substances from the brewing raw materials and contact materials that are retained or altered during subsequent processing, and new substances generated during the manufacturing processes.

#### 2.2.1. Raw Materials and Contact Materials

Raw materials for brewing ABs differ between ABs, such as grains for baijiu, beer, and huangjiu and fruits for wine. Some of the ingredients themselves contain special aroma compounds that can give the final product an attractive aroma that distinguishes it from other ABs. For example, the α-acids, β-acids, and polyphenols present in hops are chemically transformed under certain conditions to produce derivatives, which give the beer a unique bitter flavor and enrich its taste [39]. Baijiu typically contains many brewing ingredients such as sorghum, rice, glutinous rice, wheat, and corn. The ratio of these raw materials has an important impact on the quality of baijiu; for example, Yin [40] et al. studied the flavor substances of seven different multi-grain ratios of light-flavored baijiu and found that for the proportions of 80% sorghum, 10% corn, and 10% yellow rice, the contents of ethyl acetate, isoamyl alcohol, and isobutanol are the highest, the ester aroma is prominent, and the taste is pleasant. However, not all substances present in raw materials improve AB quality. For example, the widespread use of insecticides and fungicides during grape cultivation can result in the presence of pesticide residues in wine. These substances migrate, degrade, and are metabolized during the fermentation process, ultimately resulting in a decline in both flavor and health benefits. Residues of fungicides such as pyrimethanil, dimethomorph, and cyprodinil can significantly reduce the production of ethyl hexanoate, ethyl butyrate, isoamyl acetate, and ethyl caproate. This leads to a significant loss of fruity aroma in wine [41]. Similarly, the presence of natural toxins is also a concern. Beer seems to be the most susceptible to mycotoxin contamination due to its grain base and a lack of filtration and distillation during brewing. Studies have identified various types of aflatoxins and ochratoxin A residues in beer [42]. In addition to substances from raw materials, the contamination with substances from materials such as plastic tubes, bottle caps, and coated aluminum cans during the production of ABs, including fermentation, storage, and packaging, can also lead to a deterioration in AB quality. For example, retail beer is often packaged in aluminum cans coated with bisphenol A diglycidyl ether epoxy resins, which are formed from bisphenol A and epichlorohydrin, posing a risk of bisphenol A contamination [43]. Plastic conveyance pipes are used in the production of some varieties of baijiu, which can lead to the risk of phthalate plasticizer contamination [44]. This shows that the selection of high-quality brewing raw materials, the choice of appropriate ingredient ratios, and the use of safe contact materials are the basis for ensuring the superior flavor and safety of ABs.

#### 2.2.2. Manufacturing Processes

While the primary task in ensuring the quality of ABs is to guarantee the quality of raw materials and the safety of contact materials, improvements in subsequent processing can also compensate for flavor deficiencies in the raw materials, eliminate some hazardous factors resulting from contact materials, and endow new flavors. In baijiu, huangjiu, beer, and wine, esters are the most abundant key flavor compounds [1]. Esters are a good example for illustrating the effect of processing procedures on the quality of ABs. Esters in ABs typically originate from three sources. A small portion of esters are derived from the raw materials. Another pathway for ester synthesis involves spontaneous chemical esterification reactions. The third and most significant pathway is microbial synthesis during fermentation processes [45]. Given that a high content of ethanol is produced during the fermentation process, it can be used as a substrate for esterification reactions with acid, and the enzymatic esterification reactions using alcohols and acids as substrates during fermentation are considered to be the main ester synthetic reactions in AB fermentation over other ester synthetic reactions such as hemiacetal dehydration reactions [46]. Therefore, different types of microorganisms containing rich enzyme systems can affect the ester compounds present in the finished ABs. Baijiu, unlike most globally renowned alcoholic beverages, uses Jiuqu as the saccharifying and fermentation agent, which is rich in microbes and medium components, while other distilled liquors employ yeast. The succession of these microbial communities and their interrelated metabolic processes drive the metabolism and accumulation of flavor compounds in baijiu, thereby contributing to its unique flavor profile. Research has shown that lipases derived from *Monascus purpureus* and *Aspergillus niger* in baijiu can synthesize medium-chain and short-chain fatty acid esters using fatty acids and ethanol as substrates under aqueous conditions, thereby contributing to the synthesis of esters during baijiu fermentation [47]. In addition to the fermentation process, distillation also has a certain effect on esters. Research has shown that there are significant differences in the aromas of base whiskies produced using different distillation equipment (double distillation in pot stills and continuous distillation in tower stills). Pot-distilled whisky loses more esters, greatly affecting its fruity notes, but it maintains a better distillation aroma compared to tower-distilled whisky, and it can extract oak-derived aromatic compounds more effectively [48]. At the same time, esters can also be formed using alcohols and acids in the process of storing ABs. Research has found that the ester content in newly distilled baijiu is very low (e.g., ethyl butyrate and ethyl caproate), but this content increases during the aging process due to oxidation reactions [49]. However, not all esters produced during aging are beneficial to AB quality. For example, ethyl carbamate (EC), which is a naturally occurring byproduct of AB fermentation or storage processes, is toxic when orally ingested, as well as carcinogenic [50]. In the wine fermentation process, EC is formed via five pathways involving the reaction of urea, citrulline, carbamyl phosphate, cyanide, and 3a,6a-dimethylglycine with ethanol [51]. Studies have shown that the EC content of baijiu brewed from different varieties of raw materials in different production areas varies, with glutinous sorghum S3 corresponding to higher final EC yields than japonica sorghum S1 and S2 [52]. Cui [53] et al. found that the inoculation of *Lysinibacillus sphaericus* MT33 during fermentation could effectively reduce the content of urea and EC; the urea and EC contents were specifically reduced by 28.15% and 41.77%, respectively. Therefore, the content of ethyl carbamate in wine can be reduced by selecting excellent strains, improving fermentation materials, and optimizing fermentation conditions to control the production of precursor substances. In addition, there are many process-induced substrates such as nitrosamines, acrolein, furfural, hydroxymethylfurfural, and acrylamide, that are produced by heating, fermentation, or oxidation and result in poor AB quality [54]. The compounds in ABs, not limited to esters, are closely related to every detail of the production process, including the raw material, cultivation technology, environment, fermentation process, aging process, and storage method, and only by carefully controlling every link in the production can consistently high-quality ABs be achieved.

## 3. Microextraction in AB Quality Analysis

**Figure 2:**
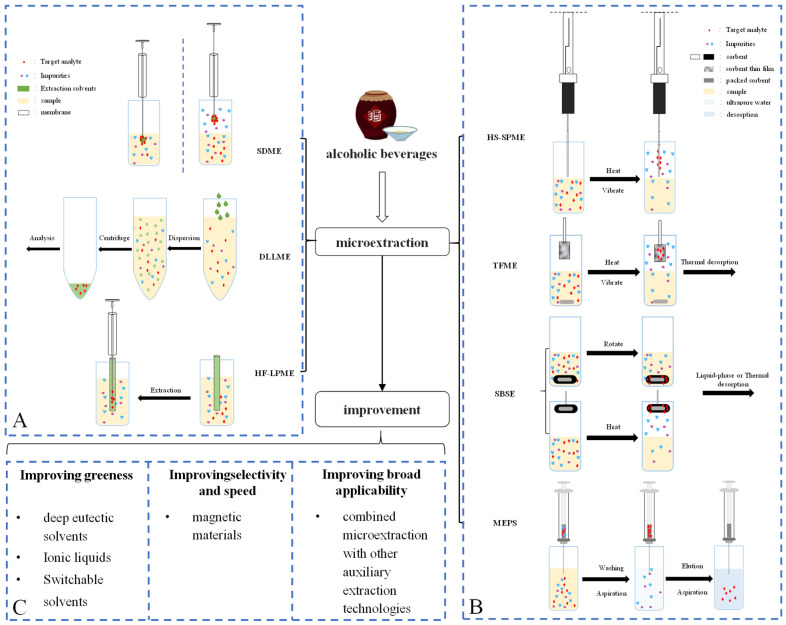
Classification, principles, and improvement of microextraction techniques. SDME: single-drop microextraction; DLLME: dispersive liquid-liquid microextraction; HF-LPME: hollow-fiber liquid-phase microextraction; HS-SPME: headspace solid-phase microextraction; TFME: thin-film microextraction; SBSE: stir bar sorptive extraction; MEPS: microextraction by packed sorbent. (**A**) The classification and principles of liquid-phase microextraction; (**B**) The classification and principles of solid-phase microextraction; (**C**) The improvement of microextraction.

AB quality is critically related to the complex compounds that determine the AB’s flavor and functional properties, and some of these compounds are often expected to be present in large quantities to enhance AB quality. However, there are some substances that are not expected to be present. The analysis of these compounds is very important for assessing the characteristics related to flavor, functional value, and safety. Sample preparation is a key step during analysis, and it can pre-concentrate and reduce complex matrix interferences. This process typically uses extraction techniques to transfer the target from the original solution to the second phase. For example, several techniques, such as solid-phase extraction (SPE), solvent-assisted flavor evaporation (SAFE), supercritical fluid extraction, purge and trap (PAT), liquid-liquid extraction (LLE), solid-phase microextraction (SPME), and dispersive liquid-liquid microextraction (DLLME), have been utilized for AB chemical analysis, and some of these methods often use large amounts of organic solvents. Today, microextraction techniques are gaining popularity because they help reduce sample or solvent utilization, allowing for the recycling of extractants and, in some cases, the use of less toxic solvents. This section describes two aspects: liquid-phase microextraction and solid-phase microextraction. An overview of their principles and development is given, followed by an introduction to their application in the quality analysis of ABs, and the general description of their principles can be found in Figure 2A,B.

### 3.1. The Application of LPME in AB Quality Analysis

Liquid-phase microextraction, an improvement of the liquid-liquid extraction method, can not only be used for the extraction of volatile, semi-volatile, polar, non-polar, and other types of compounds in ABs but also uses low sample and solvent amounts and high-power preconcentration. In addition, the analyte can be completely injected into the instrument for direct analysis, eliminating the need for other time-consuming procedures. In this section, the principle of LPME and its application in AB quality analysis are introduced. The principles of some important LPME techniques are shown in Figure 2A. LPME offers superior capabilities compared to SPME in the analysis of compounds with low volatility and water solubility. LPME enables a high analyte concentration while requiring minimal sample and extraction solvent volumes. Several LPME techniques have been applied in the analysis of ABs, although with a primary focus on pollutant determination (Table 1).

#### 3.1.1. Single-Drop Microextraction

Dasgupta [85] et al. proposed single-drop microextraction in 1996, which uses a single drop of chloroform containing methylene blue as an extractive agent suspended in a flowing sample solution to extract sodium dodecyl sulfate. Some studies have used this method to analyze health quality factors in Abs such as agricultural residues [80], cork preservatives [86], and ethyl carbamate [79]. Although SDME can rapidly and in-depth analyze alcoholic beverage samples and its enrichment effect is adequate, the stability of droplets is still the main factor limiting the development of SDME. Zaruba [87] et al. used an optical probe as a droplet holder to establish a new method for the determination of sulfur dioxide from sulfites using HS-SDME, which was applied in the analysis of food samples such as wine and juice, and its detection limit reached 8 μg/L. Although the optimization of droplet retainers has been studied, new droplet retainers are also limited to high-viscosity solvents, which limit the application of target compounds for food products with complex substrates such as ABs.

#### 3.1.2. Hollow-Fiber Liquid-Phase Microextraction

Hollow-fiber liquid-phase microextraction (HF-LPME) was proposed by Pedersen-Bjeaard [88] et al. in 1999. It is a liquid-phase microextraction method in which the extraction phase is placed in the cavity formed by the fiber membrane, which can solve the problem of stability of SDME droplets [89]. The extraction system consists of four parts: a target sample solution, hollow-fiber membrane, organic solvent filled with fiber membrane, and extraction solvent filled with fiber membrane cavity. Due to the barrier effect of the fiber membrane, this method can reduce the interference and obtain a very clean extract. The application of this method in AB analysis mainly focuses on the extraction of trace pollutants and pesticide residues. Ouyang [90] et al. used the CTC CombiPal automatic sampler to automate all extraction steps in HF-LPME, including fixing the extraction solvent, transferring and stirring the sample, drawing the solvent back into the syringe, and automatically injecting the sample into the GC system. The matrix effect in the analysis of carbamate in wine samples was corrected successfully using the HF-LPME dynamic calibration method. Bolanos [91] established an HF-LPME method that incorporated ultra-high-pressure liquid chromatography-mass spectrometry for the determination of more than 50 types of pesticide residues in wine and beer and optimized the relevant experimental parameters of microextraction. Under the optimum conditions, the detection limit (LOD) was 0.01–5.61 μg/L, with good repeatability. Romero [92] established a method for the determination of ochratoxin A and T-2 toxins in wine and beer using HF-LPME. The method used 1-octyl alcohol as the extraction solvent and ultra-high-pressure liquid chromatography combined with mass spectrometry. The relative recovery rates of this method were greater than 70%, with adequate linearity (R^2^ > 0.993). The limits of quantification (LOQs) were 0.02–0.09 μg/L. Although this method can achieve an efficient purification of the target in the complex matrix due to the strong selectivity of the membrane, the fiber membrane of the organic solvent is also a key rate-limiting step in the entire extraction process, which may lead to the blockage of the fiber membrane caused by the adsorption of non-polar impurities in the complex matrix and the blockage of some target substances by the membrane. As a result, the extraction process is slower than other microextraction technologies. In addition, in the case of non-automated operations, good repeatability of results can be achieved only when the operator is skilled [91].

#### 3.1.3. Dispersive Liquid-Liquid Microextraction

Razaee [93] established a method for extracting organic pesticides from aqueous solutions with tetrachloroethylene as the extraction agent and acetone as the dispersant in 2006, the first proposed DLLME. When compared with traditional liquid-liquid extraction, due to the addition of dispersant, the diffusion rate of organic extractant in the aqueous phase is increased, and the transfer rate of a target substance from sample solution to extractant is increased, thus reducing the amount of organic solvent and improving the extraction efficiency.

DLLME is the most commonly used method for AB analysis, compared to other LPME techniques. Several researchers have used DLLME to achieve optimized detection limits for quality factors of ABs as well as the simplification of operational methods, e.g., Fariña [94] used DLLME to detect aroma compounds in wine for the first time in 2007, using carbon tetrachloride as the extraction solvent, acetone as the dispersant, and GC-MS to detect 4-ethylguaiacol (EG) and 4-ethylphenol (EP) in wine, and their LODs were 28 μg/L and 44 μg/L, and the results showed good repeatability. Subsequently, Pizarro [95] et al. used DLLME combined with GC-MS/MS to determine the main compounds (including EG and EP) that produce a wine cork odor, and chloroform was used as the extraction agent and acetone was used as the dispersant. The LOD was 0.005~0.075 μg/L. Sun [96] et al. further optimized the method, using dichloromethane as the extraction agent and vortex extraction instead of dispersant-assisted extraction combined with GC-MS to analyze tetramethylpyrazine, 4-methyl guaiacol, and EG in baijiu, and obtained good results without using a dispersant. DLLME has several applications in the flavor analysis of ABs; e.g., Bergler [68] et al. employed DLLME combined with GC-MS to quantify nine terpenoid compounds in both wine and grape juice during fermentation. They utilized 5 mL wine samples, into which 850 μL of acetone was added as a disperser solvent, accompanied by a mere 500 μL of extraction solvent. The extraction process lasted for 20 min. The results indicated the absence of matrix effects, with LOD ranging from 5.6 to 11.3 μg/L. The method exhibited satisfactory linearity and recovery rates. A particularly interesting study is that of Sousa [73] et al., who effectively utilized DLLME coupled with GC-MS to analyze nine aromatic phenols in Portuguese red wine samples. They employed 2 mL wine samples, to which 60 μL of trichloroethylene was added as the extraction solvent alongside 500 μL of acetone as the disperser solvent. Additionally, 100 μL of derivatization reagent was added. Notably, the extraction process was remarkably swift, requiring only 5 min. Results indicated LODs ranging from 1.5 to 3.9 μg/mL, recovery rates between 69% and 113%, and excellent linearity (R^2^ > 0.992). While the DLLME method is effective, in the analysis of ABs, it is more suitable for flavor compounds with a lower volatility. For compounds with a higher volatility, solid-phase microextraction (SPME) remains more appropriate.

The predominant application of DLLME is still in the analysis of harmful substances. Abera [60] et al. developed a method combining vortex-assisted ionic liquid dispersive liquid-liquid microextraction (VA-IL-DLLME) with capillary liquid chromatography for the detection of four sulfonylurea herbicides in wine samples. They used 2.5 mL wine samples, with the ionic liquid 1-Butyl-3-methylimidazolium hexafluorophosphate as the extraction solvent and methanol as the dispersing agent, and performed extraction under 30 s of vortex assistance. Under optimal extraction conditions, the LODs and LOQs ranged from 3.2 to 6.6 μg/kg and 10.8 to 22.0 μg/kg, respectively, with RSD below 7%. Satisfactory recovery rates (79–106%) were achieved. Dal Bosco [34] et al. established a method for the determination of 19 pesticide residues in wine using a green solvent-based DLLME combined with HPLC-MS. The extraction solvent comprised a hydrophobic eutectic mixture of L-menthol and dibutyl hydroxytoluene in a molar ratio of 1:3, with the endogenous ethanol present in wine serving as the dispersing agent. The average recovery rate reached 80%, with RSD ranging from 3 to 14%. The LODs and LOQs were significantly lower than the maximum residue limits specified by EU regulations. David [69] et al. developed a method for the determination of lead and cadmium in wine using DLLME combined with electrothermal atomic absorption spectroscopy. They took 2 mL wine samples, added a buffer solution, and injected 1 mL of a mixture of 1-Butyl-3-methyl-imidazolium hexafluorophosphate (extraction solvent) and methanol (dispersing agent) using a syringe. The mixture was then subjected to vortex-assisted extraction for 2 min. The LODs for lead and cadmium were found to be 0.01 and 0.08 μg/L, respectively, which were approximately one order of magnitude lower than those reported in previous studies. Anna [67] et al. utilized DLLME combined with GC-MS to assess the levels of biogenic amines in wine. This method involved the use of a 5 mL wine sample, to which 400 μL of chloroform was added as the extractant after derivatization, followed by the addition of a mixture of 485 μL of methanol (dispersing agent), HCl, and pyridine to disperse and eliminate byproducts. Under optimal conditions, the developed method was validated, revealing excellent linearity for all compounds, with correlation coefficients ranging from 0.9961 to 0.992. The LOD was between 1.4 and 4.2 μg/L, with satisfactory recoveries (76–105%). Similarly, DLLME has been utilized for various contaminants such as agricultural residues [97], phthalates [44], mycotoxins [61], and others.

However, DLLME requires that the extractant is insoluble in water and has good enrichment, and the commonly used extractants such as carbon tetrachloride, chlorobenzene, dichloromethane, chloroform, and other halogenated hydrocarbons have certain toxicity. Moreover, one significant limitation of DLLME is the need for an extra disperser organic solvent, which often decreases the partition coefficients of the hydrophobic analytes into the extraction solvent. In order to comply with the development direction of “green chemistry” and ensure the effectiveness of the method, the improvement in the application of DLLME in ABs has focused on two main aspects: the development of green extractants and dispersants and the exploration of auxiliary extraction techniques. The first aspect encompasses the utilization of low-density extractant [98], auxiliary extractant [63], ionic liquid [97], natural deep eutectic solvent [76], and other low-toxicity extractant applications in DLLME technology, and the second aspect includes the application of device-assisted technologies (microwave, vortex, ultrasonic, mechanical agitation, etc.) [64], solvent demulsification [78], floating organic droplet solidification [82], evaporation-assisted extraction [65], etc.

### 3.2. The Application of SPME in AB Quality Analysis

Although LPME reduces the amount of extractant used and reduces environmental pollution, the best way to avoid environmental impact is to avoid the use of extractants. SPE technology meets the requirements and is appropriate for a green approach. SPE is based on selectively retaining the analyte in the adsorbent material (stationary phase) and then eluting with a solvent that shows greater affinity for the analyte, similar to the common column chromatography principles, with high selectivity. The advantages of SPE over LLE are that SPE reduces the volume of organic solvents and that extraction is faster, has greater reproducibility and repeatability, is safer for people, does not produce emulsions, and avoids the problem of incomplete phase separation. However, SPE has some drawbacks: it is relatively cumbersome, continues to use organic solvents, and consumes a large amount of sample; thus, other techniques such as solid-phase microextraction (SPME) and stir bar sorptive extraction (SBSE) have been developed. The principles of some important SPME techniques are shown in Figure 2B. We summarized the applications of various types of SPME techniques used in the quality analysis of different ABs (Table 2).

#### 3.2.1. Solid-Phase Microextraction

Solid-phase microextraction (SPME), developed by Pawlszyn [136] in 1990, is a method of extraction using the allocation of target substances between the sample matrix and the extraction medium. The extraction medium consists of fused quartz fiber coated with absorbent polymer material (stationary phase). It can be divided into headspace solid-phase microextraction (HS-SPME) and direct-immersion solid-phase microextraction (DI-SPME). The extraction time of the method for gas samples is less than that for liquid and solid samples. Therefore, HS-SPME is preferred when the target volatility is high enough or there are several impurities in the sample. In 1996, Lay-Keow [137] et al. applied SPME to ABs for the first time, using DI-SPME combined with GC-MS to investigate commercial vodka; the extracted substances included esters, aromatic active fatty acids, and furans. The primary applications of SPME lie in the field of volatile aroma compounds, but it is also used, albeit to a lesser extent, for the pretreatment of contamination.

Most AB aroma compounds have a low polarity, and the polarity of the extraction head is particularly important. Polydimethylsiloxane (PDMS) coating is a non-polar extraction phase. In order to improve the affinity for polar flavor compounds, a composite extraction phase or a medium-grade, strongly polar extraction phase is often used: polydimethylsiloxane/divinylbenzene (PDMS/DVB), divinylbenzene/carboxen/poly dimethylsiloxane (DVB/CAR/PDMS), carboxen/poly dimethylsiloxane (CAR/PDMS), polyacrylate (PA), and carbowax/divinylbenzene (CW/DVB). Metafa [138] et al. used SPME to extract aroma-active substances in wine, and used five different extraction heads, PDMS, PDMS/DVB, CAR/PDMS, DVB/CAR/PDMS, and PA, to extract them, under DI and HS modes. The optimal extraction efficiency was determined by using the PDMS/DVB extraction head in DI mode. The extraction parameters were optimized to detect 21 aroma compounds in wine. The LOQs were 1.5–30 μg/L, and the RSD was less than 21%, which could well characterize the flavor profile of the sample.

In the analysis of volatile aroma compounds in ABs, both targeted and untargeted approaches can be employed. Targeted methods aim to detect specific individual compounds or classes of compounds, whereas untargeted methods aim to comprehensively analyze the maximum number of compounds present in the alcoholic beverage. For instance, Liu [112] et al. developed an HS-SPME method combined with high-resolution gas chromatography–Orbitrap mass spectrometry (GC-Orbitrap-MS) for the determination of lactones and volatile phenols (including 12 lactones and 11 volatile phenols) in three non-grape wines. Among these compounds, volatile phenols are potential key odorants in blueberry wine, while lactones serve as indicators of high quality in hawthorn wine. The study evaluated three different fiber coatings (DVB/CAR/PDMS, CAR/PDMS, and PDMS/DVB), and the results indicated that DVB/CAR/PDMS was most suitable for the analysis of the aforementioned compound classes. Qin [107] et al. utilized HS-SPME coupled with GC-O-MS to qualitatively analyze the aroma-active compounds in three different grades of sesame-aroma baijiu. A total of 54 aroma-active compounds were identified, including 27 esters, 11 alcohols, 9 acids, 3 carbonyl compounds, 3 pyrazines, 5 furans, 2 phenols, and 3 aldehydes. Subsequently, direct injection coupled with GC-MS and LLE combined with GC×GC-SCD was employed for the quantitative analysis of these aroma compounds. The results demonstrated this method’s ability to accurately distinguish between the three different grades of baijiu. While reviewing the literature on volatile and flavor compounds in food, it becomes apparent that the frequency of solid-phase microextraction (SPME) as a quantitative method is considerably lower [139]. Some authors have highlighted the challenges associated with quantitative analysis as the main drawback of this technique [140]. Nowadays, the development and availability of internal standards, model wine calibration, stable isotope dilution analysis, matrix-matched calibrations, and standard addition contribute to eliminating matrix effects while quantifying volatile compounds, thus overcoming these limitations. However, to quantify VOCs, it is crucial to select appropriately optimized methods and suitable internal standards [141]. In this context, an interesting study conducted by Milheiro [101] et al. demonstrated the development and validation of a precise and accurate quantitative method called multiple headspace solid-phase microextraction (MHS-SPME), which eliminates matrix effects. This method was used for the quantitative analysis of volatile components in white and tawny port wine. Following the validation of methodological parameters, the correlation coefficients for all compounds ranged between 0.997 and 0.999. The obtained LODs and LOQs were both lower than the odor thresholds reported in the literature for 25% ethanol, allowing for the precise quantification of 23 aroma compounds in yellow and white port wines. These compounds include 5 acids, 14 esters, and 4 monoterpenes. In addition to flavor analysis, SPME has also been applied to functional substances. For example, Aresta [142] et al. used SPME combined with LC-UV/FID to detect trans-resveratrol in wine, spirits, and grape juices, using polyacrylate as the coating material. Under optimal conditions, accurate and efficient results were obtained. In different food matrices, the LODs were between 0.5 and 1.1 ng/mL, and LOQs were between 1.6 and 3.7 ng/mL, with high recovery rates (from 92.2 ± 4.9% to 99.4 ± 0.7%). Also, SPME has been used in the pretreatment of harmful substances. Zhou [99] et al. developed a method based on TF-SPME for high-throughput, rapid, and automated analysis of mycotoxins in beer using matrix-compatible SPME blades combined with LC-MS. This SPME system consists of multiple SPME blades with thin coating, capable of automatically processing 96 samples simultaneously. The method demonstrated good sensitivity, with LODs ranging from 0.02 to 3 ng/mL and recoveries between 79% and 121%.

SPME also has its drawbacks. In HS-SPME mode, the volatile matrix of the alcohol body and the substance to be measured are jointly vaporized, competing for the adsorption position of the fiber, thus affecting the extraction efficiency. In DI-SPME mode, the extraction head is immersed in the matrix to contact non-volatile substances, which increases the matrix effect. Moreover, its coating is thinner and its enrichment ability is weaker than that of LLE. Although SPME is suitable for the analysis of volatile and semi-volatile substances, its application has certain limitations due to the existence of non-volatile components in alcoholic beverages. Therefore, many studies have combined SPME with other extraction technologies to improve its enrichment effect [143].

#### 3.2.2. Stir Bar Sorptive Extraction

SBSE was proposed by Baltussen [144] et al. in 1999. In this method, the surface of the glass is coated with an adsorbent material and wrapped inside a magnetic rod, and the target sample solution is stirred to make the target object adsorbed to the adsorbent coating on the surface. After desorption separation, the target is detected. This method is an improvement of SPME, with a larger extraction phase volume than that of SPME fibers, and not only has the same advantages as SPME but also has higher sensitivity in the extraction of trace compounds [145]. It includes two extraction modes: headspace-type and immersion-type extraction. Unlike HS-SPME, where the adsorbent remains static throughout the extraction process, SBSE involves dynamic extraction. In this process, the adsorbent moves through the solution via magnetic force, facilitating interactions between analytes and the adsorbent. As a result, the required extraction time is typically much shorter than SPME, and sometimes even higher sensitivity is achieved. For example, Wang [146] et al. utilized comprehensive two-dimensional gas chromatography–time-of-flight mass spectrometry (GC×GC-TOF-MS) to reveal the extraction efficiency of different sample pretreatment methods (HS-SPME, SPE, and SBSE) for herbaceous aroma-type baijiu. They accurately identified 247 compounds. The results demonstrated that SBSE exhibited higher analytical sensitivity and achieved lower detection and quantification limits.

SBSE is a common technique for AB quality analysis, which is widely used in aroma compounds and contaminants. Weldegergis [147] et al. used SBSE and GC-MS to analyze 39 volatile components in Pinotagi wine; the LOD was 50 pg/L~281 ng/L, and the RSD value was less than 20%. Perestrelo [148] compared the extraction performance of SBSE and HS-SPME methods for higher alcohol acetate, isoamyl ester, and ethyl ester in wine. SPME and SBSE identified and quantized 16 and 25 esters, respectively, and SBSE had better sensitivity and a shorter extraction time. Niu [123] et al. used SBSE to characterize some volatile compounds in baijiu, with LODs ranging from 0.007 μg/L to 17.89 μg/L, and RSD was all less than 10%. They used this method to identify five different types of baijiu and a total of 87 volatile compounds were identified. Currently, the commonly used and commercialized SBSE coatings include polydimethylsiloxane (PDMS) and ethylene glycol (EG). Although PDMS is stable and can be used many times without degradation, it can only adsorb non-polar compounds. Although EG can adsorb polar substances, it has the drawbacks of a short shelf-life, instability, and weak PDMS rods. At present, in the application of SBSE in ABs, some solutions have been derived to solve the polarity problem of PDMS rods, such as derivatization, dual-stir bar sorptive extraction (Dual-SBSE) [119], sequential-stir bar sorptive extraction (Sequential SBSE) [120], and multiple stir-bar sorptive extraction (mSBSE) [117]. Maria [119] et al. developed a novel method to study the flavor profile of wine using a new dual-stir bar sorptive extraction (one stir bar placed in the headspace of the sample bottle, and another immersed in the sample). This method combined adsorptive extraction with thermal desorption and GC-MS analysis for extracting volatile compounds from wine samples. Compared to conventional DI-SBSE and HS-SBSE methods, this new approach effectively extracted various volatile and semi-volatile compounds with sufficient sensitivity for accurate identification. A total of 205 metabolites were identified.

#### 3.2.3. Thin-Film Microextraction

Thin-film microextraction (TFME) was proposed by Bruheim [149] in 2003 to fix the PDMS film on a stainless steel rod for sample extraction. Compared with SPME, TFME has a larger surface area and phase volume and is more sensitive, which means it can extract a large number of target substances in a short time [145,150]. Compared to SBSE, the film is composed of a variety of adsorbents, which solves the disadvantages of SBSE coatings in terms of polarity. Deng [151] et al. established a method combining TFME with surface-enhanced Raman scattering (SERS) for the detection of food additive SO_2_ in wine, by optimizing the experimental conditions, and they showed a good linear relationship in the range of SO_2_ concentration from 1 to 200 μg/mL, and the LOD was 0.1 μg/mL. Martyna [105] et al. established a TF-SPME method for the sequential extraction of compounds in beer by using two thin-film materials with different selectivities (PDMS/HLB (hydrophobic equilibrium adsorbent) and PDMS), and the results showed that sequential thin-film extraction could detect more substances than single thin-film extraction, and the RSD was less than 8%. There are few studies on the application of TFME to AB samples, and TFME is still in the stage of in-depth study and wide application.

#### 3.2.4. Quick, Easy, Cheap, Effective, Rugged, Safe Method

QuEChERS (quick, easy, cheap, effective, rugged, safe) is a sample pretreatment method developed by Anastassiades [152] in 2003 based on dispersed solid-phase extraction (DSPE), and the treatment process of this method is usually as follows: A dehydrating agent is added to the sample liquid to promote salt-out stratification, and an extraction agent is added to extract the target substance. After centrifugation, the upper extraction liquid is extracted, adsorption particles are added for purification, and the co-extraction matrix is then removed for analysis to reduce the matrix effect. According to the properties of the detected object, the corresponding extraction agent is selected for extraction, and the appropriate adsorbent is selected for purification. Compared with traditional LLE, a lower amount of extraction solvent is used to avoid the loss of low-boiling point compounds during solvent evaporation.

This method is mainly used for the detection of pesticide residues, toxins, and process-induced pollutants (polycyclic aromatic hydrocarbons [153], etc.) in ABs. Magdalena [132] et al. established a method for the detection and analysis of 131 pesticide residues in wine and grapes using QuEChERS combined with GC-μECD/NPD, and acetonitrile was used for extraction, and NaCl, disodium hydrogen citrate, sodium citrate dihydrate, and magnesium sulfate were used as salt-out agents to achieve phase separation. The results showed that the recovery rate of most analytes ranged from 70% to 120%, RSD ≤ 20%, and all analytes showed a good linear relationship in the range of 0.002 to 4.158 mg/kg. Kosma [129] et al. used QuEChERS combined with Ultra performance liquid chromatography orbitrap mass spectrometer to detect multiple pesticide residues in white and red wine. They extracted them with a mixture of acetonitrile, acetic acid, and magnesium sulfate and purified them with PSA and magnesium sulfate; the results showed that the LODs of 38 pesticides were 0.5~22 μg/kg, the LOQs were 1.4~73 μg/kg, the recoveries were 71.2~125%, and the RSD was less than 10%. This is an effective method for the detection of agricultural residues in wine.

At present, QuEChERS has been applied in many studies for the detection of alcohol pollutants, in which acetonitrile [44,154] and ethyl acetate [155,156] are usually used as extractants. PSA [157], PSA/GCB (graphitized carbon black) [154], PSA/C18 (octadecylsilane) [44], PSA/GCB/C18 [157], or PSA combined with new materials [158] are used as purification adsorbents.

#### 3.2.5. Microextraction by Packed Sorbent

Microextraction by packed sorbent (MEPS) is a novel sample preparation procedure developed by AbdelRehim in 2003 [159] and is the miniaturization of the traditional SPE technology. In the MEPS process, a small amount of solid adsorbent material or extractant is placed in a tight syringe for sample processing; after the extractant/adsorbent is absorbed, washed, and eluted, the syringe is connected to the instrument for automatic sampling. This is a simple, fast, and fully automated method. Compared with SPE, the sample volume and extraction solvent amount are greatly reduced, and the MEPS column can be reused hundreds of times. MEPS is carried out in a closed system, preventing the loss of analytes. At present, this method has been applied to the analysis of agricultural residues [135], bioactive substances [160], volatile substances [161], and other substances present in ABs. Leca [135] et al. developed MESP using a hand-held automated analytical syringe combined with GC-MS to detect ethyl carbamate in wine. Under optimal conditions, the LODs and LOQs were 1.5 and 4.5 ng/mL, respectively, with recovery rates ranging from 97% to 106%. When applied to 16 different wine samples, it demonstrated good selectivity.

### 3.3. The Application of Other Microextraction Techniques in AB Quality Analysis

Pacheco and coworkers [162] introduced an innovative technique known as gas-diffusion microextraction (GDME) for the first time for extracting vicinal diketones in beer. This method utilizes a unique extraction device, which features a compact Teflon tube equipped with a small microporous hydrophobic semipermeable membrane that embodies a thin air space between a donor and an acceptor solution. This membrane effectively prevents solvent diffusion while permitting the mass transfer of volatile and semi-volatile analytes in the vapor form. The extraction device is suspended or submersed in the sample, and the vial is sealed. The derivatizing agent is dissolved in the extraction solvent, which is placed inside the extractor. Subsequently, the sample is heated and agitated continuously to volatilize the target analytes, allowing them to migrate through the semipermeable membrane into the extraction solvent [163]. This method integrates derivatization and extraction and has been widely used in the extraction and analysis of aldehydes in ABs. Aldehydes are often present in trace amounts in alcoholic beverages and can be masked by peaks from high-concentration components during GC-MS analysis. Additionally, some unsaturated aldehydes are difficult to analyze due to the position of their carbon-carbon double bonds, often requiring derivatization before qualitative and quantitative analyses [164]. Ferreira [165] et al. adopted GDME for the simultaneous extraction and derivatization of aldehydes from beer using the derivatizing agent for aldehydes, i.e., 4-hydrazinobenzoic acid. Lima [166] et al. successfully achieved the high-performance liquid chromatography analysis of low-molecular-weight aldehydes, using 4-aminobenzoic acid as the derivatization reagent combined with GDME, with LOD lower than 0.5 mg/L. This method was also applied to the analysis of formaldehyde and acetaldehyde in various ABs.

## 4. Improvements Based on Commonly Used Microextraction Techniques

### 4.1. Improving the Greenness of Microextraction Using Green Solvents

Although liquid-phase microextraction methods use a lower amount of toxic solvents, they still possess certain health risks; thus, many studies have been devoted to the greenization of extraction solvents. Therefore, some new green solvents, such as deep eutectic solvents (DESs) and ionic liquids (ILs), have been developed.

DESs are eutectic mixtures composed of hydrogen bond donors and hydrogen bond acceptors with a certain molar ratio. They have lower melting points than single components and can be used to extract targets with different properties by adjusting the type and proportion of components. At present, DESs have been combined with SDME, SPME, DLLME, and other microextraction technologies and have been widely used in environmental, clinical, and food analyses for the extraction of various organic and inorganic chemicals, drugs, pesticides, and proteins. In ABs, DESs are often used for the extraction of pesticide residues. In a study performed by Zhang [132] et al., a DLLME method based on DES (hexafluoroisopropanol/menthol/thymol) elution was developed for the simultaneous extraction and preconcentration of aliphatic aldehydes from drinking water and alcoholic beverage samples before their determination by HPLC–ultraviolet. The effective parameters were optimized, and under optimal statuses, linear ranges of 1.0–200, 0.5–200, 0.2–200, 0.4–400, 1.0–400, 0.4–400, and 0.4–400 μg/L for seven aliphatic aldehydes (R^2^ ≥ 0.9949) were obtained, as well as a good precision (RSD < 4.9%) and low LODs (0.1–0.5 μg/L). In order to enhance the efficiency of DES-based DLLME, Ji [38] et al. used ultrasonic agitation to increase the dispersion of DESs (lactic acid/trioctylmethylammonium chloride) in the sample solution for the extraction and preconcentration of trace cadmium (Cd) and arsenic (As) in wine. The results showed good linear relationships (0.50–8.0 μg/L for Cd, 2.0–50 μg/L for As), and the LODs of Cd and As were 0.080 and 0.30 μg/L. In another study by Jia [82] et al., solidified DESs (thymol/octanoic acid) were investigated as the extraction solvents for effervescence tablet-assisted microextraction (ETA-ME) using HPLC to determine picoxystrobin, pyraclostrobin, and trifloxystrobin in water, juice, wine, and vinegar samples. Under optimal parameters, the LOD ranged from 0.15 to 0.38 μg/L, and extraction recovery ranged from 77.4 to 106.9%.

### 4.2. Improving the Selectivity and Speed of Microextraction Using Magnetic Nanomaterials

**Figure 3:**
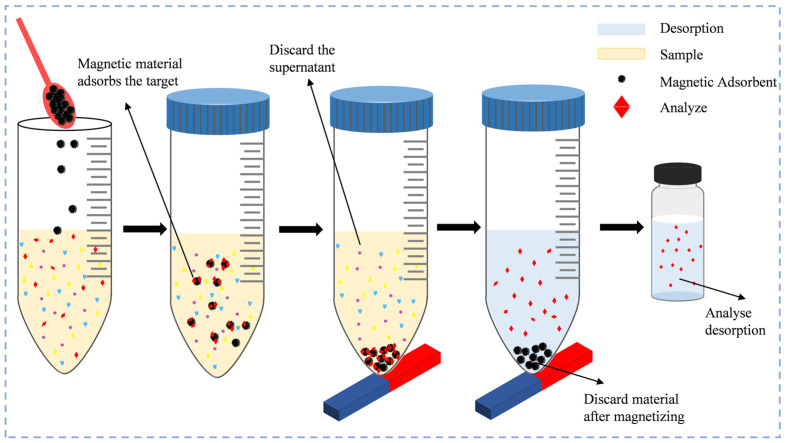
The principle of MSPE.

In addition to improving the environmental friendliness of the extractants used, the development of new adsorbents has had a significant impact on the advancement of analytical methods such as dispersive solid-phase extraction (DSPE), resulting in new methods such as magnetic solid-phase extraction (MSPE) and magnetic solid-phase microextraction (MSPME). DSPE consists of dispersing the adsorbent in the sample matrix, using the adsorbent particles to enrich the analyte, and separating the target substance from the solid-phase material using solvent elution. When the amount of adsorption material is in the milligram range, it is called dispersive micro-solid-phase extraction (D-μSPE), and sometimes, it is also called dispersive solid-phase microextraction [167]. MSPE is a new D-μSPE technique developed by SAafarikova [168] et al. in 1999, which can be thought of as dispersive solid-phase microextraction using magnetic adsorbents (Figure 3). The introduction of magnetic materials simplifies the recovery of adsorbents in D-μSPE, which is often its most complex step. At present, a variety of magnetic materials have been thoroughly studied, and new magnetic nanomaterials can be prepared according to the properties of the target substance to be tested to improve the selectivity of MSPE.

MSPE has been used for the extraction of contaminants and flavor substances in ABs. Tian [169] et al. prepared Fe_3_O_4_/CP[5]A hybrid nanomaterials based on Fe3O4 for the determination of seven trace pesticides in wine and juice samples, with LODs ranging from 5.0 to 11.3 ng/mL, and the recovery rates of all analytes ranged from 70.6% to 106.8%, and the RSD was less than 8.0%. In another study, Xu [170] et al. prepared a new spherical magnetic COF material Fe_3_O_4_@TAPT-TFTA-COF using carboxylated Fe_3_O_4_ as the magnetic core. By combining with HPLC-MS/MS, they achieved the high-efficiency enrichment and detection of benzoylurea insecticides in yellow wine and fruit juice. Low LODs (0.4–4.0 ng/L) and LOQs (1.4–13.3 ng/L) were obtained. The recoveries were 82-114%, and all RSDs were lower than 11%. Ao [171] et al. synthesized a magnetic graphene oxide nanomaterial based on polyacrylamide-modified GO/PAM/Fe_3_O_4_, and used it as an MSPE adsorbent, combined with GC-MS, to detect six odor-active esters (OAEs) in Luzhou-flavored baijiu. Under optimal parameters, the LOD ranged from 0.08 to 1.35 μg/L, and the extraction recovery ranged from 70.1 to 90.0%. Ye [172] et al. developed a method for analyzing long-chain fatty acid ethyl esters (LCFAEEs) in baijiu using MSPE combined with GC-MS and stable isotope dilution analysis. They synthesized a simple and cost-effective Fe_3_O_4_@NH_2_ adsorbent that can simultaneously extract eight types of LCFAEEs from baijiu.

Compared with other microextraction technologies, MSPE is rarely applied in AB quality analysis. Due to its high efficiency, greenness, and high selectivity to target objects, MSPE has a good application prospect in the complex matrix of ABs. For specific flavor substances and pollutants in ABs, selective magnetic nanomaterials need to be further developed.

### 4.3. Improving Applicability Using Multiple Combined Technologies

Due to the presence of very trace amounts, the complexity of the matrix, and the wide variety of compounds with different properties in ABs, it is sometimes difficult to extract these substances using a single microextraction technique. Therefore, some studies have combined microextraction with other auxiliary extraction technologies to extract trace components.

In order to simultaneously extract multiple compounds with different properties, some studies combine multiple methods to achieve comprehensive material extraction. Gong [173] et al. combined solvent-assisted flavor evaporation (SAFE) with headspace solid-phase microextraction (HS-SPME) to extract volatile flavor components from fermented grains, and the results showed that SAFE had a better extraction effect on alcohols and fatty acids, while HS-SPME had a better extraction effect on esters. In combination, the two methods have good complementarity and can achieve a more comprehensive extraction of the volatile components in fermented grains. In addition to the comprehensive extraction of substances, multivariate combinations can also enable the extraction of trace substances while avoiding complex matrix interference. For example, many studies use SPE for preconcentration before microextraction to avoid matrix interference. Feng [143] et al. established a new method based on SPE combined with HS-SPME for the determination of terpenes in baijiu. The filler in the SPE column is used to extract terpene compounds in baijiu, and the filler is then loaded into the headspace bottle for enrichment using HS-SPME. Under optimal conditions, four substances were randomly selected for methodological verification, and the LOD was found to be 0.24–6.69 μg/L. This method was used to detect terpene compounds in three typically flavored baijiu samples, and a total of 37 terpene compounds were detected, which is a new and feasible method for detecting terpene compounds in baijiu. Also, Picard [121] proposed a dual extraction method that first utilizes SPE for extraction and then employs another method for enrichment. This method, combining SPE and SBSE, can achieve rapid, convenient, and almost fully automated extraction of eight mint aroma compounds in red wine. After the optimization of conditions, LODs and LOQs of most compounds were between 2 and 45 ng/L and between 8 and 150 ng/L. Fontana [174] et al. established a method based on SPE and DLLME combined with GC-QTOF-MS/MS to determine the aroma compounds alkyl methoxypyrazines (MPs) in wine. First, SPE was used to separate the target analytes from the matrix components, followed by DLLME for analyte concentration. Under the final working conditions, the LODs were between 0.3 and 2.1 ng/L, with relative recoveries ranging from 84 to 108%. This method achieved sufficiently low LOQs, with concentrations reaching or falling below the sensory detection thresholds for wine.

## 5. Conclusions

ABs are among the most widely consumed drinks in the world, making their quality control crucial. Given that the quality factors of ABs typically exist in trace amounts, ensuring efficient extraction and analysis while adhering to the principles of green development is a significant challenge. In this context, miniaturized extraction techniques offer an ideal solution due to their sensitivity, simplicity, and high efficiency. Microextraction is applicable to a variety of compounds with different structures and polarities, reduces the use of organic solvents, and can sometimes eliminate the need for filtration and centrifugation. Although current configurations are still in the improvement stage, advancements in automated sampling provide a solid foundation for future development. Research on novel adsorbent materials and nanomaterials has driven innovations in traditional analytical chemistry methods, particularly in the rapid, selective, and sensitive removal and detection of target analytes. The environmentally friendly characteristics of microextraction make it especially important in the context of sustainable development, as it effectively reduces the consumption of organic solvents and thus lowers analytical costs while improving efficiency. Microextraction techniques still have a few drawbacks that need addressing through further improvements, such as reductions in the complexity of handling large amounts of samples; improvements in the degree of automation and integration; the development of more stable and environmentally friendly extraction media; improvements to the stability and reproducibility of the extraction process; the development of new adsorbents that are highly targeted and can be synthesized in a stable manner; and further improvements to the accuracy of SPME quantification. Looking ahead, the development of microextraction technology will focus on material innovation, the integration of various microextraction configurations, and their combination with new analytical methods. Through these ongoing advancements, microextraction will play a significant role in improving sensitivity, efficiency, and environmental friendliness of analyses, providing more reliable solutions for the assessment of AB quality and, more broadly, food quality control.

## Figures and Tables

**Table 1:** The liquid-phase microextraction methods for the analysis of quality factors in ABs.

Type of LPME	Sample	Quality Factor	Analyte	Extractant	Ext. Solvent Volume	Sample Volume ^a^	Extract Time ^b^	Auxiliary Equipment	Detection System	LOD	LOQ	Linear Range	RSD/%	Recovery /%	Ref.
VS-LLME	Baijiu	harmful components	14 Phthalate esters	tetrachloroethylene	500 μL	++	+	Emulsifier (Tween 20), vortex	GC-MS	0.05–10.0 μg/kg	0.125–20.0 μg/kg	-	0.1–6.2	83.4–122.3	[44]
	Baijiu	Aroma	roasted and mud-like aroma	CCl_4_, ether, CH_2_Cl_2_	250 μL	++	+	Emulsifier (Tween 20, Cetyl-trimethylammonium bromide), vortex	GC-MS	0.1–88.9 μg/L	-	0.005–450 μg/L	-	72–120.9	[55]
VA-LLME	Baijiu	aroma	volatile compounds	dichloromethane	1 mL	++	+	vortex	GC-MS, GC-O-MS	-	-	-	-	-	[56]
SA-LLME	wine	harmful components	biogenic amines	ethyl acetate	50 μL	+	++	salting-out	GC-MS	1.5–8.1 μg/L	5.0–27 μg/L	-	2.3–10.4	84–106	[57]
LLME	beer	harmful components	biogenic amines	supramolecular solvent (mixture of 1-Dodecanol and tetrahydrofuran	600 μL	+++	++	ultrasonic–microwave synergistic	HPLC	0.004–0.06 μg/L	0.013–0.2 μg/L	0.1–2.0 × 10^5^ μg/L	1.2–2.1	96.28–102.56	[58]
VA-DLLME	beer	harmful components	phthalates and adipates.	n-hexane	200 μL	+++	+	Ethano, vortex	GC-MS/MS	0.3–1.5 μg/L	1–5 μg/L	-	1.72–20.4	-	[59]
	Wine	harmful components	four sulfonylurea herbicides	The ionic liquid (1-hexyl-3-methylimidazolium hexafluorophosphate)	80 mg	++	+	Methanol, vortex	capillary liquid chromatography (CLC)	3.2 × 10^−6^–6.6 × 10^−6^	10–22 μg/kg	11–450 μg/kg	0.6–6.9	80–106	[60]
	huangjiu	harmful components	ochratoxin A	The ionic liquid (1-hexyl-3-methylimidazolium hexafluorophosphate ([HMIM] [PF6]))	100 μL	++	++	Ethanol, vortex	HPLC	0.04 μg/L	0.1 μg/L	-	2.7–10.4	76–82.1	[61]
	wine	harmful components	6 biogenic amines	trihexyltetradecylphosphonium tetrachlorocobalt (II) [P_6,6,6,14_^+^]_2_[CoCl_4_^2−^]	20 mg	++	+++	methanol, vortex	HPLC-UV	1.3–3.9 μg/L	4.1–9.9 μg/L	10–1000 μg/L	<4.9	93.2–103.1	[62]
PCA-DLLME	Baijiu	harmful components	Triazole fungicides	green medium-chain fatty acid (decanoic acid)	175 μL	++	+	Popping candy	HPLC-DAD	8.1–11.2 μg/L	27.1–37.3 μg/L	27.1–1000 μg/L	1.1–7.1	80.8–102.5	[63]
UA-DLLME	wine	harmful components	Cd, As	deep eutectic solvent (DL-lactic acid/trioctylmethylammonium chloride-based)	400 μL	++	+++	Methanol, vortex, sonicate	FAAS	0.08, 0.3 μg/L	0.25, 1.0 μg/L	0.5–8, 2–50 μg/L	2.9–4.5	90.6–103.6	[38]
	wine	illegal additives	melatonin	dichloromethane	250 μL	++	+++	ultrasound	HPLC-DAD	0.23 μg/L	0.7 μg/L	0.70 μg/L–15 mg/L	0.4–1.1	92–103	[64]
EVA-DLLME	wine	harmful components	organophosphate insecticides (malathion, diazinon, phosalone)	hexanol and dichloromethane mixture	400 μL	+++	+	dichloromethane evaporate	HPLC-MS/MS	3 × 10^−10^–3 × 10^−7^ g/L	-	10^−9^–10^−2^ g/L	-	92–103	[65]
UD-SA-DLLME	wine	harmful components	fungicides	1-octanol	11 μL	++	++	shaker	GC-MS	0.007–0.025 μg/L	0.024–0.082 μg/L	0.05–100 μg/L	<12	83–108	[66]
DLLME	wine	harmful components	19 pesticides	L-menthol and butylated hydroxytoluene	150 μL	+++	++	endogenous ethanol	HPLC-MS	7 × 10^−10^~1.6 × 10^−6^ g/L	0.0024–5.0 μg/L	-	3–14	56–100	[34]
	wine	harmful components	biogenic amines (BAs)	chloroform	400 μL	++	++	methanol	GC-MS	1.4–4.2 μg/L	4.6–12.6 μg/L	-	4–12	76–105	[67]
	White wine	aroma	terpenes	dichloromethane	500 μL	++	+	acetone	GC-MS	5.6–11.3 g/L	18.7–37.6 g/L	10–200 μg/L	3.3–19.4	97.9–105.3	[68]
	wine	harmful components	Cd/Pb	1-Butyl-3-methyl-imidazolium hexafluorophosphate	150 mg	++	++	methanol	ETAAS	0.01/0.08 μg/L	-	-	-	96–100	[69]
	huangjiu	aroma	Higher alcohols	dichloromethane	600 μL	++	+	acetonitrile	GC-MS	0.14–1.04 mg/L	0.47–3.45 mg/L	1.39–309.8 mg/L	1.4–9.3	80–124	[70]
	beer	harmful components	Cu	CCl_4_	100 μL	++		No used	FAAS	3.2 μg/L	9.1 μg/L	-	3–16	92–116	[71]
	wine	harmful components	biogenic amines	chloroform	400 μL	++	+	methanol	GC-MS	1.1–4.1 μg/L	3.3–12.3 μg/L	-	2–13	77–105	[72]
	wine	Aroma	vinylphenols and ethylphenols	trichloroethylene	60 μL	++	+	acetone	GC-MS	1.5–3.9 μg/mL	1–5 μg/mL	-	-	70–113	[73]
	Wine	harmful components	Cu, Fe	1,2-dichlorobenzene	40 μL	+++	-	methanol	FAAS	2.4, 6.3 μg/L	7.2, 19 μg/L	-	-	89–113	[74]
	Beer, alcoholic beverage	harmful components	Aliphatic aldehydes	DES (hexafluoroisopropanol and menthol/thymol)	100 μL	++	+	acetonitrile	HPLC-UV	0.1–0.5 μg/L	0.2–1.0 μg/L	-	1.1–5.3	77.3–119	[75]
DLLME-SFO	Wine	harmful components	pesticide residues (fipronil, fipronil-sulfide, fipronil-sulfone, and boscalid)	1-dodecanol	100 μL	++	+	natural deep eutectic solvents (NADESs): glucose, anhydrous, citric acid anhydrous, lactic acid	HPLC-UV	0.8–1.3 μg/L	2.7–4.4 μg/L	2.7–200 μg/L	1.0–12.4	>80	[76]
	wine	Appearance factor, Taste, Functional components	phenolic acids (gallic acid and protocatechuic acid)	1-dodecanol	50 μL	++	++++	tetrahexylammonium bromide (ion-pairing technique)	LC using a coreshell particle column	0.005–0.1 g/L	0.01–0.30 g/L	0.01–15.00 g/L	0.18–9.33	77.2–117	[77]
SD-DLLME	wine	harmful components	30 fungicides	1-octanol	100 μL	+++	+	Acetonitrile, vortex	HPLC-MS/MS	0.03–0.06 μg/L	0.1–0.2 μg/L	-	4–22	70–117	[78]
SDME	wine	harmful components	ethyl carbamate	butyl acetate	2 μL	+	++	microsyringe	GC-MS	1.5 ng/mL	5 ng/mL	2–1000 ng/mL	<5	94.9–99.9	[79]
SDME	wine	harmful components	eighteen pesticide residues	toluene	10 μL	+++	++++	microsyringe	GC-MS	0.1–4.62 μg/L	1.78–18.6 μg/L	0.25–25 μg/L	-	5–120	[80]
HS-SDME	wine	harmful components	Methanol	N, N-dimethylformamide	2 μL	+++	++	stirring magnet	GC-FID	0.001 mg/L	0.003 mg/L	0.05–2.0 mg/L	1.9–4.7	83.99–117.24	[81]
ETA-ME	water, juice, wine, and vinegar samples	harmful components	strobilurin fungicides	Deep eutectic solvents (thymol with octanoic acid)	120 μL	++	-	effervescence tablet (sodium bicarbonate and citric acid)	HPLC	0.15–0.38 μg/L	0.49–1.25 μg/L	-	1.0–8.6	77.4–106.9	[82]
LPME	Wine	harmful components	47 multiclass pesticides	toluene	70 μL	+++	++++	vortex	GC-MS	2.29–533 ng/L	7.63–1776 ng/L	-	<10.2	81.7–119	[83]
UA-LPME	Wine and food sample	FunctionComponents, pigment	quercetin	DES (tetrabutyl ammonium chloride and ethyl glycol)	450 μL	+++	+	Tetrahydrofuran, ultrasound bath	spectrophotometry	6.1 μg/L	20 μg/L	20–850 μg/L	1.9	98.5	[84]

^a^. Sample volume: ’+’: <1 mL, ‘++’: 1–5 mL, ‘+++’: 5–10 mL; ^b^. Extract time: ‘+’: <1 min, ‘++’: 1–5 min, ‘+++’: 5–10 min, ‘++++’: >10 min. Ext. solvent volume: extract solvent volume; LOD: limit of detection; LOQ: limit of quantitation; RSD: relative standard deviation; Ref.: reference; VS-LLME: vortex-assisted surfactant-enhanced-emulsification; VA-LLME: vortex-assisted liquid–liquid microextraction; SA-LLME: salt assisted liquid–liquid microextraction; VA-DLLME: vortex-assisted dispersive liquid–liquid microextraction; EVA-DLLME: evaporation-assisted dispersive liquid–liquid microextraction; UD-SA-DLLME: up-and-down shaker-assisted dispersive liquid–liquid microextraction; SD-DLLME: solvent demulsification-dispersive liquid-liquid microextraction; ETA-ME: effervescence tablet-assisted microextraction.

**Table 2:** The solid-phase microextraction methods for the analysis of quality factors in alcoholic beverages.

Type of SPME	Material	Sample	Quality Factor	Analyte	Sample Volume ^a^	Extract Time ^b^	Detection System	LOD	LOQ	Linear Range	RSD/%	Recovery/%	Ref.
High-throughput and automated SPME	HLB (co-polymer of hydrophilic N-vinylpyrrolidone and lipophilic divinylbenzene), (particle size: 5 μm) at length of 1 mm and thickness of 10 μm on both sides of the blade	Beer	Harmful components	Mycotoxins	++	+++	LC-MS	0.02–3 ng/mL	0.05–10 ng/mL	0.1–200 ng/mL	<13	79–121	[99]
HS-SPMESH	PDMS sheet (12.5 cm × 0.5 mm × 8.5 cm)	Wine	Aroma	Volatile phenols	+++	++	DART-MS	<1 μg/L	6 μg/L	6–250 μg/L	5–6	86–102	[100]
MHS-SPME	DVB/CAR/PDMS, 50/30 μm	Wine	Aroma	23 aroma compounds	++	++++	GC-MS	<1 μg/L	<1 μg/L	0.001–50 mg/L	<5	>95	[101]
ER-SPME	Anion-exchange monolith (AEM) (the pore sizes are around 450 nm)	Various aqueous and wine samples	Harmful component, flavor	F^−^, Cl^−^, NO^2−^, NO^3−^, Br^−^, BrO^3−^	++++	+++	IC/CD	0.015–1.5 μg/L	0.051–4.95 μg/L	0.1–500 μg/L	1.4–9.1	83.2–115	[102]
CF-SPME	PDMS/DVB, 65 μm	Beer	Harmful components	polycyclic aromatic hydrocarbons (PAHs), and their nitrated (nitro-PAHs) and oxygenated (oxy-PAHs) derivatives	+	+++	GC-MS	0.003–0.128 μg/L	0.011–0.427 μg/L	-	3.0–18.7	80.1–100.3	[103]
DI-HS-SPME	PDMS/DVB 75 μm, including 65 μm coating + 10 μm overcoating, length: 1 cm	wine	Wine quality	Volatile compounds	++	++	GC-MS	-	-	-	-	-	[104]
TF-SPME	PDMS was used to deplete non-polar and other compounds HLB/PDMS for the direct microextraction of the remaining compounds	Beer	-	polar and low volatility compounds	-	++	GC-MS	-	-	-	-	-	[105]
HS-SPME	PDMS, 100 μm	wine	aroma	volatile organic compounds	++	++	GC-MS	-	-	-	-	-	[106]
	divinybenzene/carboxen/polydimethy-lsiloxane (DVB/CAR/PDMS), 50/30 μm	Baijiu	aroma	aroma-active compounds	+++	+++	GC-O-MS	-	-	-	-	-	[107]
	DVB/CAR/PDMS, 50/30 μm	wine	aroma	volatile organic compounds	++	+++	GC-MS	-	-	-	-	-	[108]
	zeolitic imidazole framework-67 (thickness of the coating, which is around 15 μm)	Beer, vodka	harmful components, aroma	Some alcohols	+++	+++	GC-MS, GC-FID	0.17 μg/L	-	0.5–100.0 μg/L	6.8–9.6	67.5–108	[109]
	PDMS/DVB, 65 μm	tequila	aroma	terpenes	+	++	GC-MS	2.0–8.1 ng/mL	6.3–10.33 ng/mL	50–1000 ng/mL	<10	-	[110]
	DVB/CAR/PDMS	Baijiu	Authenticity (aroma types and geographical origin)	volatile compounds	+	+	GC-MS/MS	-	-	-	-	-	[111]
	DVB/CAR/PDMS, 50/30 μm	nongrape wine	aroma	lactones and volatile phenols	++	++	GC-Orbitrap-MS	0.003–37.44 μg/L, 0.02–104.28 μg/L	0.01–124.8 μg/L, 0.05–347.6 μg/L	-	8.2–18.56	80–119	[112]
	DVB/CAR/PDMS, 50/30 μm	Baijiu	Authenticity (age-markers)	Volatile compounds	++	+++	GC-MS	-	-	-	-	-	[113]
	DVB/CAR/PDMS, 50/30 μm	Wine	harmful components, taste	9 Multihalo- Phenols and Anisoles	++	+++	GC-MS/MS	3–30 ng/L	10–100 ng/L	10–10,000 ng/L	2.8–19.4	75.2–119.8	[114]
	DVB/CAR/PDMS, 50/30 μm	Wine	Authenticity (geographical origin)	volatile fraction	++	++	FM GC×GC-TOFMS	-	-	-	-	-	[115]
SPME	DVB/PDMS, 65 μm	Baijiu	Authenticity	volatile components	-	+	GC-MS	-	-	-	-	-	[116]
multi-SBSE	PDMS and polyethyleneglycol-modified silicone (EG-Silicone), 1 cm × 1 mm	botrytized wines	Authenticity (geographic origin)	volatile organic compounds	++	++++	GC-GC	-	-	-	-	-	[117]
dual sequential-SBSE	PDMS	Wine	Flavor	volatile composition	+++	+++	GC-MS	-	-	-	-	-	[118]
	PDMS (10 mm × 0.5 mm)	wine	Flavor	volatile and semivolatile compounds.	+++	++++	GC-MS	-	-	-	-	-	[119]
	PDMS (10 mm × 0.5 mm)	wine	aroma	volatile compositions	+++	+++	GC-MS	-	-	-	-	-	[120]
SPE+SBSE	PDMS (20 mm × 1 mm; length film thickness)	wine	aroma	limonene-derived monoterpenes	++++	+++	GC-MS	2–45 ng/L	8–150 ng/L	-	3.9–18.1	83–120	[121]
SBSE with thermal desorption	PDMS (10 mm × 0.5 mm)	huangjiu	Authenticity (Geographic Origin and Age	volatile compounds	+++	++	GC-MS	-	-	-	-	-	[122]
	PDMS (10 mm × 0.5 mm × 24 μL)	Baijiu	aroma	Volatile Compounds	+++	++++	GC-MS	0.007–17.89 μg/L	0.02–69.6 μg/L	-	0.2–7.0	76.3–105.6	[123]
SBSE	PDMS, 10 mm	wine	aroma	Methoxypyrazines	++	++	GC-MS/MS	0.25 ng/L	0.5 ng/L	-	0.44–19	92–108	[124]
	ethylene glycol-silicone (EG)	Brandy	Taste, aroma	Lactones	+++	+++	GC×GC-TOFMS	-	-	-	-	-	[125]
	PDMS, 10 mm/0.5 mm	medicinal liquor	aroma	Volatile compounds	++++	+++	GC-MS	-	-	-	-	-	[126]
	PDMS, 10 × 0.5 mm (length × film thickness)	Baijiu	Aroma, Functional components	active-aroma compounds and amino acids	++++	+++	GC-O, GC-FID, GC-MS	-	-	-	-	-	[127]
QuEChERS	Extract: ethyl acetate Cleanup: Primary secondary amine (PSA), 40 μm	wine	harmful components	13 fungicide residues	+++	++	LC-MS/MS	0.0003 mg/kg	0.001–0.003 mg/kg	1–50 ng/mL	3.45–6.14	80.56–97.85	[128]
	Extract: acetonitrile Cleanup: PSA, 40 μm	wine	harmful components	pesticide residues	+++	++	UHPLC-Orbitrap-MS	0.7–21.5 μg/kg	2.5–72 μg/kg	-	<11	70–120	[129]
	Extract: 10 mL acetonitrile containing 1% (*v*/*v*) acetic acid Cleanup: PSA (125 mg) and C18 (250 mg)	wine	harmful components	97 pesticides	++	+	UHPLC-MS/MS	3.0-6.0 μg/L	10-20 μg/L	-	<20	70–120	[130]
	Extract: acetonitrile containing 1% (*v*/*v*) acetic acid Cleanup: 50 mg C_18_	Beer	harmful components	Pesticides	+++	++	GC-MS/MS	0.0001–0.0007 μg/mL	0.001–0.006 μg/mL	0.001–2.5 μg/mL.	0.3–10.5	70–123	[131]
	Extract: 10 mL acetonitrile Cleanup: 150 mg PSA	wine	harmful components	over 131 pesticides	+++	+	GC-μECD, GC-NPD	-	0.009–0.023 mg/kg	0.009 and 0.023 mg/kg	≤20	72–113	[132]
MEPS	Extract:PEP (Polar Enhanced Polymer) Elution:100 μL of 50% MeOH.	wine	aroma	Sotolon	+	-	UHPLC-PDA	0.45–2.51 μg/L	1.49–8.36 μg/L	10–800 mg/L	<5.6	>81	[133]
	Extract: C8 Elution: 200 μL MeOH: H_2_O (95:5, *v*/*v*)	wine	harmful components	furanic derivatives	+	-	UHPLC-PDA	4.5–129.3 ng/L	14.9–431.0 ng/L	-	<5	74–97	[134]
	Extract: C8 Elution: 100 μL dichloromethane	wine	harmful components	ethyl carbamate	+	-	GC-MS	1.5 μg/L	4.5 μg/L	5–400 μg/L	<7	97–106	[135]

^a^. Sample volume: ’+’: <1 mL, ‘++’: 1–5 mL, ‘+++’: 5–10 mL, ‘++++’: > 10 mL; ^b^. Extract time: ’+’: <10 min, ‘++’: 10–30 min, ‘+++’: 30–60 min, ‘++++’: >60 min. LOD: limit of detection; LOQ: limit of quantitation; RSD: relative standard deviation; Ref.: reference; HS-SPMESH: headspace solid-phase microextraction sheets; MHS-SPME: multiple headspaces solid-phase microextraction; ER-SPME: electric field-reinforced solid-phase microextraction; CF-SPME: cold fiber solid-phase microextraction; TF-SPME: two thin-film solid-phase microextractions; multi-SBSE: multi-stir bar sorptive extraction; DART-MS: direct analysis in real time mass spectrometry; IC/CD: ion chromatography with conductivity detector; FM GC×GC-TOFMS: flow-modulated comprehensive two-dimensional gas chromatography with time-off light mass spectrometry; UHPLC-Orbitrap-MS: ultra-high performance Liquid Chromatography Orbitrap Mass Spectrometry; UHPLC-PDA: ultra-high pressure liquid chromatography with photo-diode detection.

## Data Availability

No new data were created or analyzed in this study. Data sharing is not applicable to this article.

## Supplementary Materials

The following supporting information can be downloaded at: https://www.mdpi.com/article/10.3390/foods14071152/s1, Table S1: The flavor, functional and harmful substances in ABs [23,31,55,175,176,177,178,179,180,181,182,183,184,185,186,187,188,189,190,191,192,193,194,195,196,197,198,199,200,201,202,203,204,205,206,207,208,209,210,211,212,213,214,215,216,217,218,219,220,221,222,223,224,225].

## Abbreviations

The following abbreviations are used in this manuscript:

ABs	Alcoholic beverages
USD	United States dollar
OAV	Odor activity value
LC-MS	Liquid chromatograph mass spectrometer
GC-MS	Gas chromatograph mass spectrometer
NMR	Nuclear magnetic resonance
FT-IR	Fourier transform infrared spectrometer
EU	Europäische Union
LODs	Limits of detection
LOQs	Limits of quantitation
RSD	Relative standard deviation
VOCs	volatile organic compounds
LC-UV	Liquid chromatograph ultraviolet and visible spectrum
LC-FID	Liquid chromatograph flame ionization detector
GC-μECD	Gas chromatograph micro electron capture detector
UHPLC-Orbitrap-MS	Ultra performance liquid chromatography orbitrap mass spectrometer
GC-NPD	Gas chromatograph nitrogen-phosphorus detector
