# Supplementary material for: Applications of Microextraction Technology for the Analysis of Alcoholic Beverages Quality: Current Perspectives and Future Directions
Source: Foods. 2025 Mar 26;14(7):1152. doi: 10.3390/foods14071152 (PMC11988442; doi:10.3390/foods14071152)
Supplement: Supplementary file 1 [file foods-14-01152-s001.zip › foods-3539924-supplementary.pdf]

Supplementary Materials

Applications of Microextraction Technology in Alcoholic Beverages Quality Analysis: Current Perspectives and Future Directions

Yue Qiu[a, b, c], Qi Deng [a, b, c], Yongqing Zhang [a, b, c], Baoguo Sun [a, b, c], Wenxian Li [a, b, c],

Wei Dong [a, b, c *], Xiaotao Sun [a, b, c, *]

a. Beijing Laboratory of Food Quality and Safety, Beijing Technology and Business University, Beijing, 100048, China.

b. Key Laboratory of Brewing Molecular Engineering of China Light Industry, Beijing Technology and Business University, Beijing, 100048, China.

c. Key Laboratory of Geriatric Nutrition and Health Ministry of Education, Beijing Technology and Business University, Beijing, 100048, China.

*Corresponding author

Wei Dong

School of Food and Health, Beijing Technology and Business University, Beijing 100048, P. R. China.

*E-mail: 20200812@btbu.edu.cn.

Xiaotao Sun

School of Food and Health, Beijing Technology and Business University, Beijing 100048, P. R. China.

*E-mail: sxt_btbu66@163.com.

Table S1. The flavor, functional and harmful substances information in alcoholic beverages

In this appendix, we summarize 130 flavor compounds with OAV values greater than 1, 139 functional substances, and 182 substances harmful to human health found in alcoholic beverages.

If you want to check whether a substance in alcoholic beverages has flavor or health effects, you can find its quality contribution in the table by searching for CAS. Through "The types of ABs", we can know which types of alcoholic beverages the substance appears in; Through "Quality Influence", we can determine which aspects of the quality of alcoholic beverages are affected by the substance; Through the "Flavor description" and "OAV", we can know which types of aroma the flavor compound contribute to, as well as their odor activity value in alcoholic beverages. Through the "Health related description", we can know what benefits or harms the substance has for the human body.

Table S1. The flavor, functional and harmful substances information in ABs

	Compound	CAS	The types of ABs	Quality influence	Flavor description	OAV	Health-related description	Reference
**Esters**	Ethyl acetate	141-78-6	Wine, baijiu, huangjiu	Aroma, functional	Fruit, solvent, sweet	2-99	Restrict the effect of acetaldehyde; accelerate the body's metabolism; anti-inflammatory dilate blood vessels; have anti-inflammatory and vasodilative functions	[175] [176] [55] [177] [178] [179] [180] [181] [182]
	Isoamyl acetate	123-92-2	Wine, huangjiu, beer, baijiu	Aroma	Banana, fresh	2-542.9	-	[175] [178] [179] [180] [181] [182] [183] [184] [185] [186]
	Ethyl lactate	97-64-3	Baijiu, wine, huangjiu	Aroma, functional	Fruity, buttery, dairy	1-38	The ethanol stimulates the cerebral cortex to produce a euphoric effect; anti-inflammatory and vasodilator; have anti-inflammatory and vasodilative functions	[176] [177] [178] [179] [180] [181] [184]
	Ethyl butyrate	105-54-4	Wine, baijiu, huangjiu, beer	Aroma	Acid fruit	2.4-8618	-	[175] [176] [55] [177] [178] [179] [180] [181] [182] [184] [185] [187]

Ethyl hexanoate	123-66-0	Wine, baijiu, beer, huangjiu	Aroma, functional	Green apple, pineapple	2-81928	Lowering lung fire and stabilizing the heart and lungs; can stabilizie heart and lung	[175] [176] [177] [178] [179] [180] [181] [182] [184] [185] [186] [187] [188]
Ethyl caprylate	106-32-1	Wine, baijiu, huangjiu, beer	Aroma	Fruit, Sweet, soap, banana, grape	2-21313	-	[175] [176] [55] [177] [178] [179] [180,184] [181] [182] [183] [185] [187]
Ethyl isovalerate	108-64-5	Baijiu, wine, huangjiu	Aroma	Citrus, pineapple	1-6484	-	[55] [178] [179] [180] [181] [182] [184] [187]
Ethyl isobutyrate	97-62-1	Baijiu, wine, beer	Aroma, functional	Citrus, apple-like	3-650	Antimicrobial activity	[55] [179] [180] [181] [182] [184] [185] [187]
Ethyl 4-methylpentanoate	25415-67-2	Baijiu	Aroma	Fruit, apple-like	101-155	-	[55]
Ethyl decanoate	110-38-3	Wine, baijiu	Aroma	Fruity, fatty, Pleasant, soap, floral	1-13	-	[175] [55] [177] [179] [183] [184]

Ethyl 2-methylbutanoate	7452-79-1	Baijiu, beer, wine	Aroma	Fruit, pineapple	5-950	-	[55] [181] [182] [187] [188]
Ethyl phenylacetate	101-97-3	Baijiu, huangjiu	Aroma, functional	Rose perfume	1-11	Antimicrobial activity	[176] [178] [180]
Ethyl 3-phenylpropionate	2021-28-5	Baijiu	Aroma, functional	Flower	5-35	Antimicrobial activity	[176] [55]
Phenethyl acetate	103-45-7	Baijiu, beer	Aroma	Smoky	1-5	-	[176] [55] [185] [186]
Hexyl hexanoate	6378-65-0	Baijiu	Aroma	Green, fruity	4-27	-	[55] [177] [180]
Ethyl pentanoate	539-82-2	Baijiu, wine	Aroma	Apple, pineapple	3-8750	-	[176] [55] [177] [179,180] [181] [182]
Butyl hexanoate	626-82-4	Baijiu	Aroma	Fruity	4-113	-	[176] [177]
Butyl butanoate	109-21-7	Baijiu	Aroma	Fruity	218-310	-	[176] [177]
Ethyl heptanoate	106-30-9	Baijiu	Aroma, functional	Pineapple	2-29	Hydrolyzed in vivo to produce fatty acids, which can inhibit cholesterol synthesis.	[176] [55] [177]
Isopentyl hexanoate	2198-61-0	Baijiu	Aroma	Pineapple, banana	7-71	-	[176] [177]
Pentanoic acid, 2-hydroxy-4-methyl-, ethyl ester	10348-47-7	Baijiu	Aroma	Fruity	5	-	[176]
Ethyl dodecanoate	106-33-2	Baijiu	Aroma	Fruity	9-17	-	[176] [180]
Propyl hexanoate	626-77-7	Baijiu	Aroma	Sweet, fruity, grape	1-3	-	[176] [55] [177]

Sotolon	28664-35-9	Beer, huangjiu, wine	Aroma	Caramel, burnt, sugar	1-11	-	[178] [185] [187] [188] [189]
Ethyl 3-phenylpropionate	2021-28-5	Baijiu, wine	Aroma, functional	Honey	1-85	Antimicrobial activity	[177] [179] [182]
Ethyl benzoate	93-89-0	Baijiu	Aroma, functional	Fruity	1	Antimicrobial activity	[177]
Butyl formate	592-84-7	Baijiu	Aroma	Fruity	64-89	-	[177]
Hexyl butyrate	2639-63-6	Baijiu	Aroma	Sweet	38	-	[177]
Ethyl nonanoate	123-29-5	Baijiu	Aroma	Grape	1	-	[177]
Ethyl hexadecanoate	628-97-7	Baijiu	Aroma	Coffee, nutty	10-376	-	[177] [180]
Ethyl propanoate	105-37-3	Huangjiu, baijiu, wine	Aroma	Sweet, banana, fruity	1.3-73	-	[178] [180] [187]
Ethyl vanillate	617-05-0	Huangjiu	Aroma	Vanilla	1.1	-	[178]
2-methylpropyl acetate	110-19-0	Baijiu	Aroma	Fruity	1-4	-	[179]
Ethyl myristate	124-06-1	Baijiu	Aroma	Sweet, waxy, violet orris	2-4	-	[180]
Propyl acetate	109-60-4	Baijiu	Aroma	Fruity, sweet	1-2	-	[180]
Ethyl hexadecenoate	68862-27-1	Baijiu	Aroma	Fruity, creamy	1-2	-	[180]
Ethyl linoleate	544-35-4	Wine, baijiu	Functional	-	-	Lowering blood cholesterol and blood lipids, preventing atherosclerosis	[190] [191] [192]

	Linolenic acid ethyl ester	1191-41-9	Baijiu	Functional	-	-	Inhibits cholesterol synthesis	[192] [193]
	Higher fatty ethyl esters	-	Baijiu	Functional	-	-	Prevention of Alzheimer's disease	[190]
	Vanillate esters	-	Baijiu	Functional	-	-	Antioxidant, anti-tumor, hypoglycemic effect, improving the body's immune system	[194]
	Alpha-Angelica lactone	591-12-8	Baijiu	Functional	-	-	Tumor biotherapy	[190]
	Linalyl acetate	115-95-7	Baijiu	Functional	-	-	Anti-ulcer effect, eases pain.	[195]
**Alcohols**	Isobutanol	78-83-1	Wine, baijiu, huangjiu, beer	Aroma, harmful	Fusel, Ethereal, grass	1-330	Limited internal security, triggering headaches	[175] [176] [177] [178] [179] [180] [181] [182] [185] [187] [196] [197]
	Isoamyl alcohol	123-51-3	Wine, baijiu, beer	Aroma, functional	Bitter, harsh, Fruit, banana	1-54	Antimicrobial activity	[175] [176] [55] [177] [184] [187] [189] [196]
	2-Phenylethanol	60-12-8	Beer, huangjiu, wine	Aroma, functional	Floral, roses	1-100	Antimicrobial activity	[175] [178] [179] [182] [183] [185] [188] [189] [196] [198]
	1-Butanol	71-36-3	Baijiu, beer, wine	Aroma, harmful	Sweet	2-121	Limited internal security, triggering headaches	[176] [55] [179] [180] [181] [197]

1-Propanol	71-23-8	Baijiu, wine	Aroma, harmful	Fruit, banana, fermented grain	1-17	Limited internal security, triggering headaches	[176] [177] [180] [181] [182] [197]
2-Butanol	78-92-2	Baijiu	Aroma	Fruity	1-3	-	[176] [177] [180]
1-Pentanol	71-41-0	Wine, baijiu	Aroma, harmful	Soap, sweet	3-9	Limited internal security, triggering headaches	[176] [177] [197]
2-Pentanol	6032-29-7	Baijiu	Aroma, harmful	Green	1	Limited internal security, triggering headaches	[177] [197]
1-Hexanol	111-27-3	Baijiu, wine	Aroma, harmful	Grass, grain	1-39	Limited internal security, triggering headaches	[176] [55] [177] [179] [180] [181] [197]
2-Furanmethanol	98-00-0	Baijiu	Aroma	Rose	5	-	[176]
4-Ethylphenol	123-07-9	Baijiu, wine	Aroma	Smoky	3-16	-	[176] [187]
4-Methylphenol	106-44-5	Baijiu	Aroma	Cheese	2	-	[176]
3-(Methylthio)-1-propanol	505-10-2	Wine, beer	Aroma, functional	Boiled, potato, rubber	3-54	Lower blood sugar	[183] [185] [187] [188] [190] [191] [192,194]
1-octanol	111-87-5	Baijiu	Aroma	Oil, citrus	2	-	[55]
(R)-Linalool	126-91-0	Beer	Aroma	Floral	1.2	-	[189]
FARNESOL	4602-84-0	Wine	Aroma, functional	Floral	1-4.9	Antimicrobial activity, Antioxidant activity, anti-ulcer effect and pain relief.	[184] [195]

2-Methyl-1-butanol	137-32-6	Baijiu, beer, wine	Aroma	Banana, cocoa, nutty	2-130	-	[177] [180] [182] [187] [198]
2-Hexanol	626-93-7	Baijiu	Aroma	Fruity	16	-	[177]
2-Heptanol	543-49-7	Baijiu	Aroma	Fruity	2	-	[177]
3-Methylbutanol	30899-19-5	Huangjiu, beer, baijiu	Aroma	Alcoholic, nail polish	1-58	-	[178] [179] [180] [181] [185]
Geosmin.	19700-21-1	Huangjiu, baijiu	Aroma	Mold, earthy	1-11	-	[178] [179]
Linalool	78-70-6	Beer	Aroma	Flower, citrus	11-76	-	[185] [199]
1-octen-3-ol	3391-86-4	Baijiu	Aroma	Mushroom	4-17	-	[179] [181]
(2R,3R)-(-)-2,3-Butanediol	24347-58-8	Baijiu	Aroma	Celery, fruity	2-5	-	[180]
(Z)-4-heptenal	6728-31-0	Beer	Aroma	Grass, grease	4	-	[198]
Geraniol	106-24-1	Wine	Aroma, functional	Floral	1	-	[182]
Glycerol	56-81-5	Wine, beer, baijiu	Functional	-	-	Emollient, anti-caries disease, prevention of constipation and diabetes effects	[190] [194]
Mannitol	87-78-5	Baijiu, beer, wine	Functional	-	-	Preventing early acute renal insufficiency; preventing rise in blood pressure, arteriosclerosis	[190]

Cyclohexane-1,2,3,4,5,6-hexol	6917-35-7	Wine, baijiu	Functional	-	-	Treatment of cirrhosis, fatty liver, hepatitis, high blood cholesterol	[190]
Xylitol	87-99-0	Beer, wine, baijiu	Functional	-	-	Prevention of dental caries	[190]
Sorbitol	50-70-4	Beer, wine, baijiu	Functional	-	-	Prevents the rise of blood pressure, arteriosclerosis, etc.	[190] [200]
Dulcitol	608-66-2	Beer, wine, baijiu	Functional	-	-	Preventing constipation; preventing dental caries, preventing diabetes	[190] [192] [194]
Erythritol	149-32-6	Beer, wine, baijiu	Functional	-	-	Emollient, anti-caries disease, prevention of constipation and prevention of diabetes effects	[190] [192] [194]
Propylene glycol	57-55-6	Beer, baijiu	Functional	-	-	Moisturizing, preventing dental caries, preventing constipation and preventing diabetes	[194]
Ethanol	64-17-5	Beer, wine, baijiu	Functional	-	-	Antiseptic and anesthetic, treat chronic digestive diseases, insomnia	[190] [201]
Isopropyl alcohol	67-63-0	Baijiu	Harmful	-	-	Limited internal security, triggering headaches	[197]
1-Heptanol	111-70-6	Baijiu, wine	Harmful	-	-	Limited internal security, triggering headaches	[197]

**Acids**	Butyric acid	107-92-6	Wine, baijiu, huangjiu, beer	Aroma，functional	Cheese	1-307	Inhibit the growth and reproduction of tumor cells, promote the death of tumor cells, and induce the transformation of cancer cells into normal cells	[175] [176] [55] [177] [178] [179] [180] [181] [182] [184] [185] [190] [191] [193]
	Isovaleric acid	503-74-2	Wine, baijiu, beer, huangjiu	Aroma	Blue cheese, dairy	1-93	-	[175] [176] [55] [177] [178] [179] [181] [182] [183] [185] [188]
	Hexanoic acid	142-62-1	Wine, baijiu, huangjiu	Aroma，functional	Cheese, fatty	1-447	Function of inhibiting cholesterol synthesis	[175] [176] [55] [177] [178] [179] [180] [181] [182] [183] [192] [201]
	Octanoic acid	124-07-2	Wine, baijiu	Aroma，functional	Rancid, harsh, cheesy, fatty	1.6-10.4	Function of inhibiting cholesterol synthesis	[175] [176] [55] [177] [182] [183] [192] [201]
	Decanoic acid	334-48-5	Wine	Aroma, functional	Fatty	1.3-3	Function of inhibiting cholesterol synthesis	[175] [182] [201]
	2-Methylpropionic acid	79-31-2	Baijiu, huangjiu	Aroma	Acidic, cheesy	1-49	-	[176] [55] [177] [178] [179] [180] [181]

Acetic acid	64-19-7	Baijiu, wine, beer, huangjiu	Aroma, functional	Vinegar, acidity	1-140	Inhibit germs, help digestion; lower cholesterol, lower blood fat	[176] [55] [177] [178] [179] [180] [181] [184] [185] [187] [188] [190] [191] [193]
Pentanoic acid	109-52-4	Baijiu	Aroma	Cheesy, dairy	1-146	-	[176] [55] [177] [179] [180] [181]
4-Methylpentanoic acid	646-07-1	Baijiu	Aroma	Cheesy	2-9	-	[176] [55]
Heptanoic acid	111-14-8	Baijiu	Aroma，functional	Honey	1-2	Inhibiting cholesterol synthesis	[55] [177] [192] [201]
3-phenyl propionic acid	501-52-0	Baijiu	Aroma	Mushroom, cinnamon	1	-	[55]
Propionic acid	79-09-4	Baijiu	Aroma	Vinegar	1	-	[55]
3-Methyl pentatonic acid	105-43-1	Baijiu	Aroma	Cheesy	38	-	[177]
Nonanoic acid	112-05-0	Baijiu	Aroma	Acidity, cheese	1-2	-	[177] [180]
Phenylacetic acid	103-82-2	Beer, wine	Aroma	Honey, bees-wax	4-14	-	[187] [188]
2-methylbutanoic acid	600-07-7	Wine	Aroma	-	2	-	[187]
Ferulic acid	1135-24-6	Baijiu	Functional	-	-	Antioxidant, antithrombotic, antiinflammatory, analgesic, treatment of coronary heart disease	[190] [194] [196] [202] [23]
Lactic acid	50-21-5	Beer, wine, baijiu	Functional	-	-	Prevent cellular aging, and inhibit harmful pathogenic bacteria	[190] [191] [193]

Propionic acid	79-09-4	Wine, baijiu	Functional	-	-	Prevent cellular aging, inhibit harmful pathogenic bacteria, inhibit colonic inflammation	[196]
Linoleic acid	60-33-3	Beer, wine, baijiu	Functional	-	-	Prevent cardiovascular and cerebrovascular diseases, antioxidant, delaying aging	[190] [191] [192]
Succinic acid	110-15-6	Wine, baijiu	Functional	-	-	Inhibit bacteria; enhance body immunity	[190]
Phytic acid	83-86-3	Wine, baijiu	Functional	-	-	Treatment of cirrhosis, hepatitis, fatty liver, high blood cholesterol	[190]
Calcium phytate	3615-82-5	Wines, baijiu	Functional	-	-	Treatment of various diseases of the nervous system	[190]
D(-)-Tartaric acid	526-83-0	Wine, beer, baijiu	Functional	-	-	Antiinflammatory and antiviral; increases central nervous system excitability	[190] [193]
L-Malic acid	97-67-6	Wine, baijiu	Functional	-	-	Relieve cough and asthma; treat anemia, uremia, low immunity, high blood pressure	[190] [193]
Linolenic acid	463-40-1	Beer, baijiu	Functional	-	-	Enhance immune function, inhibit cancer, lower blood lipid levels, improves vascular	[190] [191] [193] [192]

							health, anti-inflammatory	
	Fatty acids	9150-89-4	Baijiu	Functional	-	-	Preventing Alzheimer's Disease	[190]
	Palmitic acid	57-10-3	Beer, Wine, baijiu	Functional	-	-	Inhibits cholesterol synthesis	[191]
	Oleic acid	112-80-1	Beer, Wine, baijiu	Functional	-	-	Inhibits cholesterol synthesis	[191] [192]
	6-Aminocaproic acid	60-32-2	Baijiu	Functional	-	-	Antimicrobial activity	[203]
	L-Lactic acid	79-33-4	Baijiu	Functional	-	-	Promotes bifidobacteria growth, maintains micro-ecological balance. Inhibits bad bacteria, supports digestion, boosts immunity, and prevents cellular aging.	[191]
	4-Aminobutyric acid	56-12-2	Wine, baijiu	Functional	-	-	Sedative, hypnotic, anti-anxiety and anti-epileptic agent	[190]
	Propionic	123-62-6	Beer, Wine, baijiu	Functional	-	-	Regulate calorie intake, reduce cholesterol levels	[204]
**Phenols**	P-cresol	106-44-5	Baijiu, wine, beer	Aroma	Smoke, narcissus	2.4-20	-	[55] [177] [184] [188]
	4-Ethylguaiacol	2785-89-9	Baijiu, huangjiu	Aroma，functional	Bacon, grass, wood	1-98	Reduces blood sugar, prevents heart disease, antioxidant, anti-inflammatory	[176] [55] [178] [177] [190] [191] [194] [195]

							[200] [23] [201] [205] [206]
4-methyl guaiacol	93-51-6	Baijiu	Aroma，functional	Smoky	1	Antioxidant, anti-inflammatory	[55] [200] [201] [23] [205] [206]
4-Vinylguaiacol	7786-61-0	Beer, wine, huangjiu	Aroma，functional	Smoke, sweet, phenolic	1.19-20	Antioxidant, anti-tumor, improve the body's immune system	[178] [184] [185] [189] [191] [194] [198]
2,6-Dimethoxyphenol	91-10-1	Wine	Aroma	Nut, smoke	1-2	-	[184]
2-methoxyphenol	90-05-1	Beer, huangjiu, baijiu, wine	Aroma，functional	Smoky, sweet	1-67	Treat cough in chronic bronchitis	[178] [181] [188] [190] [191] [194] [196] [198]
Maltol	118-71-8	Beer	Aroma	Caramel	3		[188]
Eugenol	97-53-0	Beer, wine, baijiu	Aroma，functional	Clove	3	Antipyretic, analgesic, anti-inflammatory, anticancer	[188] [190] [195] [196]
Phenol	108-95-2	Huangjiu	Aroma	Phenolic, medicinal	1-6	-	[178]
4-vinylphenol	2628-17-3	Beer, wine	Aroma	Smoky, leather-like	1-11	-	[182] [185]
3,4,5-trihydroxystilbene	501-36-0	Wine, baijiu	Functional	-	-	Vasorelaxation, Anti-inflammation, anti-oxidation, anti-atherosclerosis, improving The lipid profile	[23] [207]

	Tannins	-	Wine	Functional	-	-	Antioxidant, anti-inflammatory	[208] [209]
	Vanillic acid	121-34-6	Beer，wine	Functional	-	-	Antioxidant, anti-tumor, hypoglycemic effect	[191] [194]
	Thymol	89-83-8	Beer, baijiu	Functional	-	-	Antiseptic; expectorant; antioxidant	[190] [195] [196]
	ERIODICTYOL	552-58-9	Baijiu	Functional	-	-	Antioxidant, hypoglycemic and hypolipidemic	[210]
	Catechins	7295-85-4	Huangjiu	Functional	-	-	Reduce oxidative stress, inflammation, protecting against heart disease and hypertension, regulate blood pressure.	[211]
	Gallic acid	149-91-7	Beer, wines, baijiu	Functional	-	-	Antioxidant, anti-apoptotic, cardioprotective, neuroprotective, and anticancer	[204]
	L-Epicatechin	490-46-0	Beer, wine, baijiu	Functional	-	-	Prevent cardiovascular diseases.	[192]
	(-)-epigallocatechin	970-74-1	Baijiu	Functional	-	-	Prevent cardiovascular diseases.	[192]
**Aldehydes and ketones**	Nonanal	124-19-6	Wine, baijiu	Aroma	Apple, raspberry, strawberry	1-13.9	-	[179] [183]
	Decanal	112-31-2	Baijiu, wine	Aroma	Orange, skin-like	2.7-11	-	[179] [183]

Furaneol(4-hydroxy-2,5-dimethyl-3(2H)-Furanone)	3658-77-3	Beer, baijiu	Aroma, functional	Caramel, sour-sweet	2.59-45	Antioxidant and anti-cancer activities	[185] [188] [189] [201]
1-Octen-3-one	4312-99-6	Beer	Aroma	Nut, mushroom	2.54	-	[189]
2,3-Butanedione	431-03-8	Wine, beer	Aroma	Butter	1.8-23	-	[184] [187] [198]
Β-Damascenone	23696-85-7	Wine, beer, baijiu	Aroma	Candy	36-900	-	[179] [181] [182] [184] [185] [186] [188] [198]
Phenylacetaldehyde	122-78-1	Beer, huangjiu, baijiu	Aroma	Honey	1-29	-	[178] [179] [181] [188] [198]
2′-aminoacetophenone	551-93-9	Beer	Aroma	Foxy	5-13	-	[185] [188]
Benzaldehyde	100-52-7	Huangjiu, baijiu	Aroma	Almond	2-5	-	[178] [179]
Vanillin	121-33-5	Huangjiu, beer, baijiu	Aroma, functional	Sweet, vanilla	5-59	Antioxidant, anti-tumor, improve the body's immune system	[178] [185] [191] [201] [194] [205] [23] [206]
Acetaldehyde	75-07-0	Beer, baijiu, wine	Aroma	Pungent	21-351	-	[180] [182] [185] [186] [198]
3-methylbutanal(3-Methylbutyraldehyde)	590-86-3	Beer, baijiu	Aroma	Grass, malt	37-671	-	[180] [181] [185] [198]
1,1-diethoxyethane	105-57-7	Beer, baijiu	Aroma	Fruity	1-347	-	[179] [180] [181] [185]

Hexanal	66-25-1	Beer, baijiu, wine	Aroma, harmful	Grassy, green	8-32	May be a health hazard	[179] [181] [212]
2-Methylbutyraldehyde	96-17-3	Baijiu	Aroma	Grass, plant, malt	267-437	-	[180]
3-Hydroxy-2-butanone	513-86-0	Baijiu	Aroma	Fatty, buttery, sweet	97-174	-	[180]
Isobutyraldehyde	78-84-2	Baijiu, beer	Aroma	Pungent, malt, green	6-121	-	[180] [198]
2-pentanone	107-87-9	Baijiu	Aroma	Wine	26-89	-	[180]
Β-Ionone	79-77-6	Baijiu	Aroma, functional	Floral	1-3	In vitro anti-tumor cell activity	[181] [195]
Methional	3268-49-3	Beer	Aroma	Toast, potato	181	-	[198]
5-ethyl-4-hydroxy-2-methyl-3(2H)-furanone	27538-09-6	Beer	Aroma	Caramel, fruity	3	-	[198]
Undecan-4-olide	104-67-6	Wine	Aroma	Peaches	2	-	[182]
Quercetin	117-39-5	Wine	Functional	-	-	Anti-atherosclerotic, anti-hypertensive, and vasodilatory activities	[213] [214]
Xanthohumol	6754-58-1	Beer	Functional	-	-	Anti-cancer, anti-atherosclerotic, And anti-obesity agents	[215] [216] [217]
Pulegone	89-82-7	Wine, baijiu	Functional	-	-	Antimicrobial activity	[195]
Fenchone	7787-20-4	Baijiu	Functional	-	-	Antimicrobial activity	[195]
Thujone	546-80-5	Baijiu	Functional	-	-	Antimicrobial activity	[195]

	Tannins	1401-55-4	Beer, wine, baijiu	Functional	-	-	Antioxidant and anti-inflammatory properties	[23]
	Anthocyanins	528-58-5	Wine, baijiu	Functional	-	-	Antioxidants	[23]
	2-Nonanone	821-55-6	Baijiu	Functional	-	-	Antimicrobial activity	[196]
	Butyraldehyde	123-72-8	Baijiu	Harmful	-	-	May be a health hazard	[212]
	Valeraldehyde	110-62-3	Baijiu	Harmful	-	-	May be a health hazard	[212]
**Sulfur compounds**	Dimethyl trisulfide	3658-80-8	Baijiu, huangjiu, beer, wine	Aroma, functional	Cabbage	3.4-467	One of the components of Allium cepa, with strong inhibitory effect on platelet aggregation.	[55] [178] [181] [182] [185] [191] [204]
	3-(methylthio)propanal	3268-49-3	Baijiu, beer, wine	Aroma	Cooked potato-like	2-52	-	[55] [185] [187] [188]
	Methanethiol	74-93-1	Wine, baijiu	Aroma, functional	Garlic, cheese, onion	1.2	Antioxidants	[182] [204]
	Ethyl 3-methylthioacetate	4455-13-4	Baijiu	Functional	-	-	Lower blood sugar	[190]
	Dimethyl disulfide	624-92-0	Beer, wine, baijiu	Functional	-	-	Reduces oxidative stress damage to cells	[190] [192]
	Ethyl 3-methylthiopropionate	13327-56-5	Beer, wine， baijiu	Functional	-	-	Lower blood sugar	[190] [191] [192] [194] [204]
	Furfuryl mercaptan	98-02-2	Baijiu	Functional	-	-	Antioxidant activity	[190] [192] [204]
	3-methylthiopropylamine	4104-45-4	Baijiu	Functional	-	-	Antioxidant activity	[204]
	5-Ethyl-2-methylpyridine	104-90-5	Baijiu	Functional	-	-	Antioxidant activity	[194]

**Nitrogenous**	3-methylindole	83-34-1	Baijiu	Aroma	Mouse-like, fecal	8	-	[55]
	2-acetyl-1-pyrroline	85213-22-5	Beer	Aroma	Roasty, popcorn	2-73	-	[188]
	2-ethyl-3,5-dimethylpyrazine	55031-15-7	Beer	Aroma	Earthy	8-38	-	[188]
	2-ethyl-3,6-dimethylpyrazine	13360-65-1	Beer	Aroma	Earthy	15-34	-	[188]
	2,3-diethyl-5-methylpyrazine	18138-04-0	Beer	Aroma	Earthy	1-8	-	[188]
	3-Isopropyl-2-methoxypyrazine	25773-40-4	Wine	Aroma	Peas	2	-	[182]
	3-sec-Butyl-2-methoxypyrazine	24168-70-5	Wine	Aroma	Green, bell papper	2	-	[182]
	3-Isobutyl-2-methoxypyrazine	24683-00-9	Wine	Aroma	Green papper	2	-	[182]
	2-methylpyrazine	109-08-0	Baijiu	Functional	-	-	Lowering blood lipids, preventing thrombosis, Antioxidant	[190] [192] [23] [218] [219]
	2,3,5,6-tetramethylpyrazine	1124-11-4	Baijiu	Functional	-	-	Lowering blood lipids, improving circulation, regulating lipid metabolism, and protecting cardiac myocytes	[190] [191] [192] [194] [201] [23] [218] [219] [220]
	2,3-Dimethylpyrazine	5910-89-4	Beer, baijiu	Functional	-	-	Antioxidant activity	[190] [192]
	Trimethyl-pyrazine	14667-55-1	Beer, baijiu	Functional	-	-	Antioxidant	[190] [192] [194] [201]

**Lactones**	γ -Nonanolactone	104-61-0	Baijiu, beer, huangjiu	Aroma	Sweet, milk, coconut	1-5	-	[55] [178] [179] [185] [189]
	γ -Decalactone	706-14-9	Huangjiu	Aroma	Sweet	1-2	-	[178]
	Cis-whisky lactone	39212-23-2	Wine	Aroma	Woody, Coconut	14	-	[187]
**Furans**	Furfural	98-01-1	Baijiu	Aroma	Sweet, nutty, bread	1-26	-	[176] [177] [180]
	2-Furan methanol	98-00-0	Baijiu	Aroma	Roasted sesame	1-2.6	-	[177] [180]
	2-Butylfuran	4466-24-4	Baijiu	Aroma	Oranges	1157	-	[177]
	5-ethyl-3-hydroxy-4-methyl-2(5H)-furanone	698-10-2	Beer	Aroma	Caramellic	36	-	[198]
**Heterocyclic compounds**	DAMASCONE	23726-91-2	Baijiu	Functional	-	-	Analgesic, antibacterial, antiviral, cancer prevention and anticancer	[190]
	Β -Caryophyllene	87-44-5	Beer, baijiu	Functional	-	-	Inhibits oxidative stress and inflammatory, antitumor, antiviral and anti-inflammatory	[190] [201] [221]
	2,6-Dimethylpyrazine	108-50-9	Beer, baijiu	Functional	-	-	Antioxidant activity	[194]
	Furan	110-00-9	Beer, baijiu	Functional	-	-	Antioxidant activity	[196]
	Thiophene	110-02-1	Baijiu	Functional	-	-	Antioxidant activity	[196]
	Thiazole	288-47-1	Baijiu	Functional	-	-	Antioxidant activity	[196]
	Benzofuran	271-89-6	Baijiu	Functional	-	-	Antimicrobial activity	[196]

**Terpenoids**	P-Cymene	99-87-6	Baijiu	Functional	-	-	Antibacterial activity, analgesic effect	[190] [195]
	(-)-ALPHA-CEDRENE	469-61-4	Baijiu	Functional	-	-	Antibacterial, inhibits osteoclast resorption in an osteoporosis model	[190]
	Terpinen-4-ol	562-74-3	Beer, baijiu	Functional	-	-	Antiviral, antioxidant, digestive aid	[190]
	DL-Menthol	89-78-1	Baijiu	Functional	-	-	Antipruritic and cough suppressant	[190] [195]
	Cis-Anethol	104-46-1	Baijiu	Functional	-	-	Antiviral, antioxidant, antimicrobial	[190]
	Nerolidol	7212-44-4	Baijiu	Functional	-	-	Scavenge hydroxyl radicals	[190]
	CADINENE	29350-73-0	Baijiu	Functional	-	-	Antimicrobial activity	[190]
	Geranyl acetate	105-87-3	Baijiu	Functional	-	-	Antimicrobial activity	[190]
	(+)-DELTA-CADINENE	483-76-1	Baijiu	Functional	-	-	In vitro anti-tumor cell activity	[195]
	D-CAMPHOR	464-49-3	Baijiu	Functional	-	-	High bacterial inhibitory activity, inhibited osteoclast resorption in an osteoporosis model	[195]
	Carvacrol	499-75-2	Baijiu	Functional	-	-	Antimicrobial activity, antioxidant activity	[195]
	Cinnamaldehyde	104-55-2	Baijiu	Functional	-	-	Antimicrobial activity	[195]
	Estragole	140-67-0	Baijiu	Functional	-	-	Antimicrobial activity	[195]
	1, 8-cineole	470-82-6	Beer, baijiu	Functional	-	-	Antibacterial, anticholinesterase, anti-nocicptive, anti-ulcer, relieve pain.	[195]

	Citral	5392-40-5	Baijiu	Functional	-	-	Antimicrobial activity, antioxidant activity	[195]
	Borneol	6627-72-1	Baijiu	Functional	-	-	Antimicrobial activity	[195]
	Sabinene	3387-41-5	Baijiu	Functional	-	-	Antimicrobial activity, antioxidant activity	[195]
	Limonene	138-86-3	Baijiu	Functional	-	-	Antibacterial activity with some antioxidant activity	[195]
	ALPHA-PINENE	2437-95-8	Baijiu	Functional	-	-	Antimicrobial activity	[195]
	Pinene	80-56-8	Baijiu	Functional	-	-	Antibacterial activity with some antioxidant activity	[195]
	Α-Terpinene	99-86-5	Baijiu	Functional	-	-	Antioxidant activity	[195]
	Terpinolene	586-62-9	Baijiu	Functional	-	-	Antioxidant activity	[195]
	Citronellal	106-23-0	Baijiu	Functional	-	-	Antioxidant activity	[195]
	Menthone	10458-14-7	Baijiu	Functional	-	-	Antioxidant activity	[195]
	Isomenthone	491-07-6	Baijiu	Functional	-	-	Antioxidant activity	[195]
	Tocopherol	119-13-1	Baijiu	Functional	-	-	Antioxidant activity	[195]
	Germacrened,1-methyl-5-methylene-8-(1-methylethyl)-1,6-cyclodecadiene	23986-74-5	Baijiu	Functional	-	-	Paroxysmal effects	[195]
	Turpentine oil	8006-64-2	Baijiu	Functional	-	-	Strong analgesic effect	[195]
**Others**	Phenylacetaldehyde	122-78-1	Baijiu	Aroma	Rose	6-25	-	[176] [55]
	Anthocyanins	11029-12-2	Wine	Functional	-	-	Antioxidants, protect against inflammation,	[213] [222] [223]

						Arteriosclerosis, and hypercholesterolemia	
Lovastatin	75330-75-5	Baijiu	Functional	-	-	Inhibit extracellular matrix secretion and proliferation of glomerular mesangial cells, and decreases the karyokinesis index in hepatocellular carcinoma cells.	[190]
Protocatechualdehyde	139-85-5	Baijiu	Functional	-	-	Anti-inflammatory and increases coronary blood flow	[190] [192] [195]
5-Hydroxymethylfurfural	67-47-0	Beer, baijiu	Functional	-	-	Anti-tyrosinase, tumor inhibiting, blood cholesterol lowering, anti-inflammatory, insecticidal	[190] [192]
Isoxanthohumol	521-48-2	Baijiu	Functional	-	-	Anti-cancer, anti-atherosclerotic, anti-obesity agents	[23]
8-prenylnaringenin	68682-02-0	Beer, baijiu	Functional	-	-	Anti-cancer, anti-atherosclerotic, and anti-obesity agents	[23]
Choline hydroxide	123-41-1	Baijiu	Functional	-	-	Antimicrobial	[203]
Betaine	107-43-7	Baijiu	Functional	-	-	Antimicrobial, good restorative effect on ethanol-damaged hepatocytes	[203]

	Allopurinol	315-30-0	Beer，baijiu	Functional	-	-	Antimicrobial	[203]
	D-Proline	344-25-2	Baijiu	Functional	-	-	Antimicrobial	[203]
	Adenosine	58-61-7	Beer, baijiu	Functional	-	-	Antimicrobial	[203]
	Adenine	73-24-5	Beer, baijiu	Functional	-	-	Antimicrobial	[203]
	Lichenin	215-755-3	Baijiu	Functional	-	-	Bacteriostatic, anticancer, antiviral, antioxidant, anti-inflammatory	[192] [195]
	Fengycin	102577-03-7	Baijiu	Functional	-	-	Anti-bacterial, anti-cancer, anti-virus,	[195]
	Subtilin	-	Baijiu	Functional	-	-	Anti-bacterial, anti-cancer, anti-virus	[195]
**Metals**	Arsenic compounds	7784-45-4	Wine, beer, spirits	Harmful	-	-	Oncogenic	[31]
	Cadmium	7440-43-9	Wine, liquor, beer	Harmful	-	-	Organ damage, lung and kidney dysfunction, bone fractures, prostatitis, hypertension	[197] [31]
	Copper	7440-50-8	Wine, beer	Harmful	-	-	Harmful to the body	[197] [31]
	Lead	7439-92-1	Beer, wine, spirits	Harmful	-	-	Poisoning, cumulative toxicity, organ damage, digestive system disorders	[190] [197] [31]
	Manganese	7439-96-5	Baijiu, beer, wine	Harmful	-	-	Too much can be harmful to body organs	[197]

	Iron	7439-89-6	Baijiu, beer, wine	Harmful	-	-	Harmful in excess	[197]
	Calcium	7440-70-2	Baijiu, wine, beer	Harmful	-	-	Too much can be harmful to body organs	[197]
	Chromium	7440-47-3	Baijiu, beer, wine	Harmful	-	-	Harmful to body organs; irritates and corrodes the respiratory tract, causing tuberculosis, bronchitis	[197]
	Mercury	7439-97-6	Baijiu, beer, wine	Harmful	-	-	Cumulative toxicity can damage organs, causing liver and kidney failure, brain damage, urinary incontinence, and fetal developmental issues	[197]
	Arsenic	7440-38-2	Baijiu, beer, wine	Harmful	-	-	Cause severe organ damage	[197]
	Mercury	7439-97-6	Baijiu, beer, wine	Harmful	-	-	Dimethylmercury is highly toxic, fatal on skin contact, and disrupts nutrient absorption, causing osteoporosis and bone fractures	[197]
	Aluminum	7429-90-5	Baijiu, beer, wine	Harmful	-	-	Inhibits pepsin activity and disrupts absorption of phosphorus, iron, and calcium, leading to osteoporosis and bone fractures	[197]
**Pesticides**	Azoxystrobin	131860-33-8	Wine	Harmful	-	-	May be a health hazard	[31]

Azinphos-methyl	86-50-0	Wine	Harmful	-	-	May be a health hazard	[31]
Benalaxyl	71626-11-4	Wine	Harmful	-	-	May be a health hazard	[31]
Benalaxyl-M	98243-83-5	Wine	Harmful	-	-	May be a health hazard	[31]
Boscalid	188425-85-6	Wine	Harmful	-	-	May be a health hazard	[31]
Chlorpyrifos	2921-88-2	Wine	Harmful	-	-	May be a health hazard	[31]
Chlorpyrifos-methyl	5598-13-0	Wine	Harmful	-	-	May be a health hazard	[31]
Cyprodinil	121552-61-2	Wine	Harmful	-	-	May be a health hazard	[31]
Dimethoate	60-51-5	Wine, baijiu	Harmful	-	-	Induces chronic diseases	[197] [31]
Dimethomorph	110488-70-5	Wine	Harmful	-	-	May be a health hazard	[31]
Fenarimol	60168-88-9	Wine	Harmful	-	-	May be a health hazard	[31]
Fenhexamid	126833-17-8	Wine	Harmful	-	-	May be a health hazard	[31]
Fenitrothion	122-14-5	Wine	Harmful	-	-	May be a health hazard	[31]
Fenthion	55-38-9	Wine	Harmful	-	-	May be a health hazard	[31]
Fludioxonil	131341-86-1	Wine	Harmful	-	-	May be a health hazard	[31]
Glyphosate	1071-83-6	Beer	Harmful	-	-	May be a health hazard	[31]
Haloxyfop-methyl	69806-40-2	Wine	Harmful	-	-	May be a health hazard	[31]

	Iprodione	36734-19-7	Wine	Harmful	-	-	May be a health hazard	[31]
	Iprovalicarb	140923-17-7	Wine	Harmful	-	-	May be a health hazard	[31]
	Kresoxim-methyl	143390-89-0	Wine	Harmful	-	-	May be a health hazard	[31]
	Metalaxyl	57837-19-1	Wine	Harmful	-	-	May be a health hazard	[31]
	Methidathion	950-37-8	Wine	Harmful	-	-	May be a health hazard	[31]
	Parathion-methyl	298-00-0	Wine	Harmful	-	-	May be a health hazard	[31]
	Penconazole	66246-88-6	Wine	Harmful	-	-	May be a health hazard	[31]
	Procymidone	32809-16-8	Wine	Harmful	-	-	Impact on development and reproductive health	[31]
	Propiconazole	60207-90-1	Wine	Harmful	-	-	May be a health hazard	[31]
	Pyrimethanil	53112-28-0	Wine	Harmful	-	-	May be a health hazard	[31]
	Quinalphos	13593-03-8	Wine	Harmful	-	-	May be a health hazard	[31]
	Tebuconazole	107534-96-3	Wine	Harmful	-	-	May be a health hazard	[31]
	Vinclozolin	50471-44-8（83792-61-4）	Wine	Harmful	-	-	Impact on development and reproductive health	[31]
	Dichlorvos	62-73-7	Baijiu, wine	Harmful	-	-	Inducement of chronic diseases	[197]
**Pesticides-organic chlorine**	HEXACHLOROBENZENE	118-74-1	Baijiu, beer	Harmful	-	-	Impairment of liver function, affecting	[197]

						reproduction, affecting embryonic development	
DDT	3416-05-5、50-29-3	Baijiu	Harmful	-	-	Impairment of liver function, affecting reproduction, affecting embryonic development	[197]
LINDANE	58-89-9	Baijiu, wine, beer	Harmful	-	-	Impairment of liver function, affecting reproduction, affecting embryonic development	[197]
TOXAPHENE	8001-35-2	Baijiu	Harmful	-	-	Impairment of liver function, affecting reproduction, affecting embryonic development	[197]
Malathion	121-75-5	Baijiu, beer, wine	Harmful	-	-	Difficult to degrade, can be converted to more toxic secondary pollutants	[197]
Trichlorfon	52-68-6	Baijiu, wine	Harmful	-	-	Difficult to degrade, can be converted to more toxic secondary pollutants	[197]
Phoxim	14816-18-3	Baijiu	Harmful	-	-	Difficult to degrade, can be converted to more toxic secondary pollutants	[197]
EPN	2104-64-5	Baijiu	Harmful	-	-	Acutely toxic pesticides, difficult to degrade, can be converted to more	[197]

							toxic secondary pollutants	
**Pesticides-carbamate**	Carbaryl	63-25-2	Baijiu, wine	Harmful	-	-	Mutagenic, teratogenic and carcinogenic	[197]
	METOLCARB	1129-41-5	Baijiu	Harmful	-	-	Mutagenic, teratogenic and carcinogenic	[197]
	3,4,5-TRIMETHACARB	2686-99-9	Baijiu	Harmful	-	-	Mutagenic, teratogenic and carcinogenic	[197]
	Isoprocarb	2631-40-5	Baijiu	Harmful	-	-	Mutagenic, teratogenic and carcinogenic	[197]
	CPMC	3942-54-9	Baijiu	Harmful	-	-	Mutagenic, teratogenic and carcinogenic pesticides	[197]
	Carbofuran	1563-66-2	Baijiu	Harmful	-	-	Mutagenic, teratogenic and carcinogenic	[197]
	Fenobucarb	3766-81-2	Baijiu	Harmful	-	-	Mutagenic, teratogenic and carcinogenic	[197]
**Pesticides-pyrethroid**	Deltamethrin	52918-63-5	Baijiu	Harmful	-	-	Can induce allergic asthma, easy to respiratory and circulatory failure	[197]
	Cypermethrin	52315-07-8	Baijiu	Harmful	-	-	Can induce allergic asthma, easy to respiratory and circulatory failure	[197]
	Fenvalerate	51630-58-1	Baijiu	Harmful	-	-	Can induce allergic asthma, easy to respiratory and circulatory failure	[197]

**Pesticides-benzimidazole**	Carbendazim	10605-21-7	Baijiu, wine	Harmful	-	-	Irritates eyes, respiratory system and skin, carcinogenic	[197]
	THIOPHANAT-ETHYL	23564-06-9	Baijiu, wine	Harmful	-	-	Irritates eyes, respiratory system and skin, carcinogenic	[197]
	Thiophanate-methyl	23564-05-8	Baijiu, wine	Harmful	-	-	Irritates eyes, respiratory system and skin, carcinogenic	[197]
	FUBERIDAZOLE	3878-19-1	Baijiu	Harmful	-	-	Irritates eyes, respiratory system and skin, carcinogenic	[197]
**Pesticides-herbicides**	2,4-Dichlorophenoxyacetic acid	94-75-7	Baijiu	Harmful	-	-	Disrupts the normal function of the nervous system, immunodeficiency disorders, carcinogenic	[197]
	Sodium pentachlorophenolate	131-52-2	Baijiu, beer	Harmful	-	-	Disrupts the normal function of the nervous system, immunodeficiency disorders, carcinogenic	[197]
	Paraquat methosulfate	2074-50-2	Baijiu	Harmful	-	-	Disrupts the normal function of the nervous system, immunodeficiency disorders, carcinogenic	[197]
	NITROFEN	1836-75-5	Baijiu	Harmful	-	-	Disrupts the normal function of the nervous system,	[197]

						immunodeficiency disorders, carcinogenic	
Propanil	709-98-8	Baijiu	Harmful	-	-	Disrupts the normal function of the nervous system, immunodeficiency disorders, carcinogenic	[197]
Trifluralin	1582-09-8	Baijiu, beer	Harmful	-	-	Disrupts the normal function of the nervous system, immunodeficiency disorders, carcinogenic	[197]
Glyphosate	1071-83-6	Baijiu	Harmful	-	-	Group 2A carcinogenic compounds, immunodeficiency disorders, carcinogenicity	[197]
Alachlor	15972-60-8	Beer	Harmful	-	-	Strong teratogenicity, carcinogenicity and mutagenicity	[224]
Acetochlor	34256-82-1	Beer	Harmful	-	-	Strong teratogenicity, carcinogenicity and mutagenicity	[224]
Pretilachlor	51218-49-6	Beer	Harmful	-	-	Strong teratogenicity, carcinogenicity and mutagenicity	[224]
Machette	23184-66-9	Beer	Harmful	-	-	Strong teratogenicity, carcinogenicity and mutagenicity	[224]

	Propisochlor	86763-47-5	Beer	Harmful	-	-	Strong teratogenicity, carcinogenicity and mutagenicity	[224]
	Metolachlor	51218-45-2	Beer	Harmful	-	-	Strong teratogenicity, carcinogenicity and mutagenicity	[224]
	Propanil	709-98-8	Beer	Harmful	-	-	Strong teratogenicity, carcinogenicity and mutagenicity	[224]
	Thiobencarb	28249-77-6	Beer	Harmful	-	-	Strong teratogenicity, carcinogenicity and mutagenicity	[224]
**Mycotoxins**	Aflatoxin B1	1162-65-8	Wine, beer, baijiu	Harmful	-	-	Carcinogenic, teratogenic, mutagenic	[197] [31]
	Aflatoxin B2	7220-81-7	Wine, beer, baijiu	Harmful	-	-	Carcinogenic, teratogenic, mutagenic	[197] [31]
	Aflatoxin G1	1165-39-5	Wine, beer, baijiu	Harmful	-	-	Carcinogenic, teratogenic, mutagenic	[197] [31]
	Aflatoxin G2	7241-98-7	Wine, beer, baijiu	Harmful	-	-	Carcinogenic, teratogenic, mutagenic	[197] [31]
	Aflatoxin M1	6795-23-9	Wine, beer, baijiu	Harmful	-	-	Carcinogenic, teratogenic, mutagenic	[197] [31]
	Ochratoxin A	-	Wine, beer, baijiu	Harmful	-	-	Class 2B carcinogenic compounds, teratogenic, mutagenic	[197] [31]

	Nivalenol	23282-20-4	Beer	Harmful	-	-	May be a health hazard	[31]
	Deoxynivalenol	51481-10-8	Wine, beer, baijiu	Harmful	-	-	Carcinogenic, teratogenic, mutagenic	[197] [31]
	Fumonisin B1	116355-83-0	Wine, beer	Harmful	-	-	May be a health hazard	[31]
	Fumonisin B2	116355-84-1	Beer	Harmful	-	-	May be a health hazard	[31]
	HT-2 toxin	26934-87-2	Wine, beer	Harmful	-	-	May be a health hazard	[31]
	Sterigmatocystin	10048-13-2	Beer	Harmful	-	-	May be a health hazard	[31]
	T-2 toxin	21259-20-1	Beer	Harmful	-	-	May be a health hazard	[31]
	Zearalenone	17924-92-4	Beer	Harmful	-	-	May be a health hazard	[31]
	ZEARALANONE	5975-78-0	Baijiu	Harmful	-	-	Carcinogenic, teratogenic, mutagenic	[197]
**Volatile organic compounds**	Methanol	67-56-1	Wine	Harmful	-	-	Safe within limits or cause blindness	[197] [212] [31]
	Tert-Butanol and other alcohols	75-65-0	Wine	Harmful	-	-	May be a health hazard	[31]
**Processed induced chemicals**	Ethyl carbamate	51-79-6	Wine	Harmful	-	-	Class 2A carcinogen	[197] [31]
	Acetaldehyde	75-07-0	Beer, wine	Harmful	-	-	May be a health hazard	[212] [31]
	Formaldehyde	50-00-0	Beer, wine	Harmful	-	-	Causes vomiting, diarrhea, leukemia, nasopharyngeal cancer; carcinogenic	[197] [212] [31]
	5-Hydroxymethylfurfural(HMF)	67-47-0	Beer, wine	Harmful	-	-	May be a health hazard	[31]

	Furfural	98-01-1	Wine	Harmful	-	-	May be a health hazard	[212] [31]
	Furfuryl alcohol	98-00-0	Wine	Harmful	-	-	May be a health hazard	[31]
	Acrylamide	79-06-1	Beer, wine	Harmful	-	-	Class 2A carcinogenic compounds	[31]
	Acrolein	107-02-8	Wine	Harmful	-	-	May be a health hazard	[31]
	N-Nitrosodimethylamine(NDMA)	62-75-9	Beer, wine	Harmful	-	-	Class 2A carcinogenic compounds	[31]
	N-Nitrosodiethylamine(NDEA)	55-18-5	Beer, wine	Harmful	-	-	Class 2A carcinogenic compounds	[31]
	N-Nitrosopyrrolidine(NPYR)	930-55-2	Beer	Harmful	-	-	May be a health hazard	[31]
	N-Nitrosomorpholine(NMOR)	59-89-2	Beer	Harmful	-	-	May be a health hazard	[31]
	Furan	110-00-9	Beer, wine	Harmful	-	-	Class 2A carcinogenic compounds	[31]
	1,2-Benzanthracene	56-55-3	Wine	Harmful	-	-	May be a health hazard	[31]
	Benzo[a]pyrene	50-32-8	Wine	Harmful	-	-	May be a health hazard	[31]
	Benzo[b]fluoranthene	205-99-2	Wine	Harmful	-	-	May be a health hazard	[31]
	Benzo[k]fluoranthene	207-08-9	Wine	Harmful	-	-	May be a health hazard	[31]
	1,12-Benzoperylene	191-24-2	Wine	Harmful	-	-	May be a health hazard	[31]
	Dibenzo[a,h]anthracene	53-70-3	Wine	Harmful	-	-	May be a health hazard	[31]
	Indeno[1,2,3-c,d]pyrene	193-39-5	Wine	Harmful	-	-	May be a health hazard	[31]
	Benzene	71-43-2	Beer	Harmful	-	-	Oncogenic	[31]
	4-Methylimidazole	822-36-6	Beer	Harmful	-	-	Class 2B carcinogenic compounds	[31]
	3-Monochloropropane diol	96-24-2	Beer	Harmful	-	-	Class 2B carcinogenic compounds	[31]
**Industrial contaminants**	Ortho-phenylphenol	90-43-7	Beer	Harmful	-	-	May be a health hazard	[31]
	Trihalomethanes	-	Beer	Harmful	-	-	Mutagenicity, carcinogenicity,	[31]

							teratogenicity and neurotoxic effects	
**Photoinitiators**	Benzophenone	119-61-9	Wine	Harmful	-	-	Class 2B carcinogenic compounds	[31]
	Isopropyl-thioxanthone	5495-84-1	Wine	Harmful	-	-	May be a health hazard	[31]
**Phthalates**	Di(2-ethylhexyl)phthalate	117-81-7	Wine, baijiu, beer	Harmful	-	-	Harming the immune and digestive systems, and causing cancers, malignant tumors, and teratology.	[31] [225]
	N-Dibutyl phthalate	84-74-2	Wine, baijiu, beer	Harmful	-	-	Harming the reproductive, immune, and digestive systems, and leading to cancers, malignant tumors, and teratology.	[226,225]
	Benzyl butyl phthalate	85-68-7	Wine, beer	Harmful	-	-	Causes endocrine disruption, leads to cancer, malignant tumors, and teratology	[225] [31]
	Diisobutyl phthalate	84-69-5	Baijiu, beer	Harmful	-	-	Affecting the reproductive, immune, and digestive systems, and leading to cancers, tumors, and birth defects.	[225] [31]
	Diethyl phthalate	84-66-2	Wine, baijiu, beer	Harmful	-	-	Harm reproductive, immune, and digestive systems, leading to	[226,225]

						cancer, tumors, and birth defects.	
Diisononyl phthalate	28553-12-0	Baijiu, Wine	Harmful	-	-	Damage the reproductive, immune, and digestive systems, leading to cancer, tumors, and birth defects.	[197]
Dimethyl phthalate	131-11-3	Baijiu, Wine	Harmful	-	-	Causing endocrine disruption, causing damage to the reproductive, immune and digestive systems, and leading to cancer, malignant tumors, teratology	[197]
BIS(4-METHYL-2-PENTYL)PHTHALATE	146-50-9	Baijiu, Wine	Harmful	-	-	Causing endocrine disruption, causing damage to the reproductive, immune and digestive systems, and leading to cancer, malignant tumors, teratology	[197]
BIS(2-ETHOXYETHYL)PHTHALATE	605-54-9	Baijiu, Wine	Harmful	-	-	Destroys the reproductive system; causing endocrine disruption, damage to immune and digestive systems, and leading to	[197]

						cancer, malignant tumors, teratology	
DI-N-PENTYL PHTHALATE-D4	131-18-0	Baijiu, Wine	Harmful	-	-	Destroys the reproductive system; causing endocrine disruption, damage to immune and digestive systems, and leading to cancer, malignant tumors, teratology	[197]
DI-N-HEXYL PHTHALATE	84-75-3	Baijiu, Wine	Harmful	-	-	Destroys the reproductive system; causing endocrine disruption, damage to the immune and digestive systems, and leading to cancer, malignant tumors, teratology	[197]
BIS(2-N-BUTOXYETHYL)PHTHALATE	117-83-9	Baijiu, Wine	Harmful	-	-	Destroys the reproductive system; damage to the immune and digestive systems, and leading to cancer, malignant tumors, teratology	[197]
Dicyclohexyl phthalate	84-61-7	Baijiu, Wine	Harmful	-	-	Destroys the reproductive system; damage to the immune and digestive systems,	[197]

						and leading to cancer, malignant tumors, teratology	
DIPHENYL PHTHALATE	84-62-8	Baijiu, Wine	Harmful	-	-	Destroys the reproductive system; damage to the immune and digestive systems, and leading to cancer, malignant tumors, teratology	[197]
Dinonyl phthalate	84-76-4	Baijiu, Wine	Harmful	-	-	Destroys the reproductive system; damage to the immune and digestive systems, and leading to cancer, malignant tumors, teratology	[197]
Bis(2-methoxyethyl) phthalate	117-82-8	Baijiu, Wine	Harmful	-	-	Destroys the reproductive system; damage to the immune and digestive systems, and leading to cancer, malignant tumors, teratology	[197]
DI-N-OCTYL PHTHALATE	117-84-0	Baijiu, Wine	Harmful	-	-	Destroys the reproductive system; damage to the immune and digestive systems, and leading to cancer,	[197]

							malignant tumors, teratology	
	Diallyl phthalate	131-17-9	Baijiu, Wine	Harmful	-	-	Destroys the reproductive system; damage to the immune and digestive systems, and leading to cancer, malignant tumors, teratology	[197]
**Undeclared ingredients,including allergens**	Gluten, wheat	8002-80-0	Beer	Harmful	-	-	May be a health hazard	[31]
	Sulfite	14265-45-3	Wine	Harmful	-	-	May be a health hazard	[31]
**Illegal additives and adulterants**	Monoethylene glycol(MEG)	15054-86-1	Wine	Harmful	-	-	May be a health hazard	[31]
	Artificial colorants	-	Beer	Harmful	-	-	May be a health hazard	[31]
	Cyanide	-	Baijiu, Wine	Harmful	-	-	Limited internal security, preventing normal cellular respiration and asphyxiation	[197]
**Biogenic amine**	Tryptamine	61-54-1	Baijiu	Harmful	-	-	Much can cause headache, nausea, respiratory disorders.	[197]
	2-phenylethylamine	64-04-0	Baijiu, beer, wine	Harmful	-	-	Much can cause headache, nausea, respiratory disorders.	[197]
	1,5-Diaminopentane	462-94-2	Baijiu	Harmful	-	-	Much can cause headache, nausea, respiratory disorders.	[197]

	1,4-DIAMINOBUTANE	110-60-1	Baijiu	Harmful	-	-	Much can cause headache, nausea, respiratory disorders.	[197]
	Histamine	51-45-6	Baijiu, wine, beer	Harmful	-	-	Much can cause headache, nausea, respiratory disorders.	[197]
	Tyramine	51-67-2	Baijiu, wine	Harmful	-	-	Much can cause headache, nausea, respiratory disorders.	[197]
	N1,N1'-(butane-1,4-diyl)bis(ethane-1,2-diamine)	35513-90-7	Baijiu	Harmful	-	-	Much can cause headache, nausea, respiratory disorders.	[197]
	Spermidine	124-20-9	Baijiu	Harmful	-	-	Much can cause headache, nausea, respiratory disorders.	[197]
**Sweetener (food additive)**	Acesulfame potassium	55589-62-3	Baijiu	Harmful	-	-	Excessive intake can damage human kidney function, and even cause teratogenicity, carcinogenicity, mental system disorders and liver damage	[197]
	Saccharin sodium dihydrate	6155-57-3	Baijiu	Harmful	-	-	Excessive intake can damage human kidney function, and even cause teratogenicity, carcinogenicity, mental system disorders and liver damage	[197]

Aspartame	22839-47-0	Baijiu	Harmful	-	-	Excessive intake can damage human kidney function, and even cause teratogenicity, carcinogenicity, mental system disorders and liver damage	[197]
Alitame	80863-62-3	Baijiu	Harmful	-	-	Excessive intake can damage human kidney function, and even cause teratogenicity, carcinogenicity, mental system disorders and liver damage	[197]
Neotame	165450-17-9	Baijiu	Harmful	-	-	Excessive intake can damage human kidney function, and even cause teratogenicity, carcinogenicity, mental system disorders and liver damage	[197]
Molasses	68476-78-8	Baijiu	Harmful	-	-	Excessive intake can damage human kidney function, and even cause teratogenicity, carcinogenicity, mental system disorders and liver damage	[197]
Stevioside	57817-89-7	Baijiu	Harmful	-	-	Excessive intake can damage human kidney	[197]

							function, and even cause teratogenicity, carcinogenicity, mental system disorders and liver damage	
	Sucralose	56038-13-2	Baijiu	Harmful	-	-	Excessive intake can damage human kidney function, and even cause teratogenicity, carcinogenicity, mental system disorders and liver damage	[197]

OAV: odor activity value

References

175. Gil, M.; Cabellos, J.M.; Arroyo, T.; Prodanov, M. Characterization of the volatile fraction of young wines from the Denomination of Origin “Vinos de Madrid” (Spain). *Analytica Chimica Acta*. **2006**, 563, 145-153. https://doi.org/10.1016/j.aca.2005.11.060.
176. Hong, J.; Huang, H.; Zhao, D.; Sun, J.; Huang, M.; Sun, X.; Sun, B. Investigation on the key factors associated with flavor quality in northern strong aroma type of Baijiu by flavor matrix. *Food Chemistry*. **2023**, 426, 136576. https://doi.org/10.1016/j.foodchem.2023.136576.
177. Hong, J.; Wang, J.; Zhang, C.; Zhao, Z.; Tian, W.; Wu, Y.; Chen, H.; Zhao, D.; Sun, J. Unraveling variation on the profile aroma compounds of strong aroma type of Baijiu in different regions by molecular matrix analysis and olfactory analysis. *RSC Adv*. **2021**, 11, 33511-33521. https://doi.org/10.1039/d1ra06073b.
178. Chen, S.; Xu, Y.; Qian, M.C. Comparison of the aromatic profile of traditional and modern types of Huang Jiu (Chinese rice wine) by aroma extract dilution analysis and chemical analysis. *Flavour and Fragrance Journal*. **2018**, 33, 263-271. https://doi.org/10.1002/ffj.3440.
179. Gao, W.; Fan, W.; Xu, Y. Characterization of the key odorants in light aroma type chinese liquor by gas chromatography-olfactometry, quantitative measurements, aroma recombination, and omission studies. *J Agric Food Chem*. **2014**, 62, 5796-5804. https://doi.org/10.1021/jf501214c.
180. Guan, Q.; Meng, L.; Mei, Z.; Liu, Q.; Chai, L.; Zhong, X.; Zheng, L.; Liu, G.; Wang, S.; Shen, C. Volatile Compound Abundance Correlations Provide a New Insight into Odor Balances in Sauce-Aroma Baijiu. *Foods*. **2022**, 11, 3916. https://doi.org/10.3390/foods11233916.
181. Li, H.; Zhang, X.; Gao, X.; Shi, X.; Chen, S.; Xu, Y.; Tang, K. Comparison of the Aroma-Active Compounds and Sensory Characteristics of Different Grades of Light-Flavor Baijiu. *Foods*. **2023,** 12, 1238. https://doi.org/10.3390/foods12061238.
182. Zhao, P.; Qian, Y.; He, F.; Li, H.; Qian, M. Comparative Characterization of Aroma Compounds in Merlot Wine by LiChrolut-EN-Based Aroma Extract Dilution Analysis and Odor Activity Value. *Chemosensory Perception*. **2017**, 10, 149-160. https://doi.org/10.1007/s12078-017-9236-4.
183. Jiang, B.; Xi, Z.; Luo, M.; Zhang, Z. Comparison on aroma compounds in Cabernet Sauvignon and Merlot wines from four wine grape-growing regions in China. *Food Research International*. **2013**, 51, 482-489. https://doi.org/10.1016/j.foodres.2013.01.001.
184. Li, H.; Kong, W.; Zhou, X.; Zhang, W. Analysis and evaluation of aroma components in wine brewed with new variety of Cabernet Gernischt. *China Brewing*. **2020**, 39(12), 164-170. https://doi.org/10.11882/j.issn.0254-5071.2020.12.031.
185. Langos, D.; Granvogl, M.; Schieberle, P. Characterization of the key aroma compounds in two bavarian wheat beers by means of the sensomics approach. *J Agric Food Chem*. **2013**, 61, 11303-11311. https://doi.org/10.1021/jf403912j.
186. Methner, Y.; Dancker, P.; Maier, R.; Latorre, M.; Hutzler, M.; Zarnkow, M.; Steinhaus, M.; Libkind, D.; Frank, S.; Jacob, F. Influence of Varying Fermentation Parameters of the Yeast Strain Cyberlindnera saturnus on the Concentrations of Selected Flavor Components in Non-Alcoholic Beer Focusing on (E)-beta-Damascenone. *Foods*. **2022**, 11, 1038. https://doi.org/10.3390/foods11071038.
187. Nicolotti, L.; Mall, V.; Schieberle, P. Characterization of Key Aroma Compounds in a Commercial Rum and an Australian Red Wine by Means of a New Sensomics-Based Expert System (SEBES)-An Approach To Use Artificial Intelligence in Determining Food Odor Codes. *J Agric Food Chem*. **2019**, 67, 4011-4022. https://doi.org/10.1021/acs.jafc.9b00708.
188. Fechir, M.; Reglitz, K.; Mall, V.; Voigt, J.; Steinhaus, M. Molecular Insights into the Contribution of Specialty Barley Malts to the Aroma of Bottom-Fermented Lager Beers. *J Agric Food Chem*. **2021**, 69, 8190-8199. https://doi.org/10.1021/acs.jafc.1c01846.
189. Kishimoto, T.; Noba, S.; Yako, N.; Kobayashi, M.; Watanabe, T. Simulation of Pilsner-type beer aroma using 76 odor-active compounds. *J Biosci Bioeng*. **2018**, 126, 330-338. https://doi.org/10.1016/j.jbiosc.2018.03.015.

190. Long, Y.; Tang, J.; Wang, X.; Shi, W.; Wu, D. Research progress on health factors and their enrichment pathways in Baijiu. *China Brewing*. **2021**, 41(02), 23-28. https://doi.org/10.11882/j.issn.0254-5071.2022.02.005.
191. Wu, T.; Zhu, S.; Sun, X.; Zhao, W.; Cui, G. Analysis of Health Factors of Meilanchun Sesame-flavor Liquor. *Liquor-Making Science & Technology*. **2013**, 08, 125-130. https://doi.org/10.3969/j.issn.1001-9286.2013.08.037.
192. Huo, J.; Huang, M.; Sun, B.; Zheng, F.; Sun, J.; Sun, X.; Li, H. Research Progress in Functional Factors in Baijiu. *Liquor-Making Science & Technology*. **2017**, 09, 17-23. https://doi.org/10.13746/j.njkj.2017174.
193. Xu, Z.; Chen, Y.; Zhou, Z.; Tang, Q. Study on Healthy & Functional Compositions in Jian'nanchun Liquor. *Liquor-Making Science & Technology*. **2008**, 05, 41-44.
194. Gao, C. 2017. Study on flavor compounds and biological activity of sesame flavor liquor. thesis, Hebei University Of Technology, Wuhan, China.
195. Fan, W.; Xu, Y. Review of Important Functional Compounds Terpenes in Baijiu. *Liquor Making*. **2013**, 40(06), 11-16. https://doi.org/10.3969/j.issn.1002-8110.2013.06.008.
196. Cheng, F. 2019. Study on the mechanism of Baijiu-induced liver injuryand regulation of intestinal flora based on omic approaches. Ph.D. dissertation, JiangNan university, Jiangsu, China.
197. Yan, H.; Zhao, Y.; Huan, D.; Zong, W.; Song, F. Research Progress in Analysis and Detection of Trace Harmful Components in Baijiu. *Liquor-Making Science & Technology*. **2022**, 10, 94-106. https://doi.org/10.13764/j.njkj.2022036.
198. Piornos, J.A.; Balagiannis, D.P.; Methven, L.; Koussissi, E.; Brouwer, E.; Parker, J.K. Elucidating the Odor-Active Aroma Compounds in Alcohol-Free Beer and Their Contribution to the Worty Flavor. *J Agric Food Chem*. **2020**, 68, 10088-10096. https://doi.org/10.1021/acs.jafc.0c03902.
199. Takoi, K.; Koie, K.; Itoga, Y.; Katayama, Y.; Shimase, M.; Nakayama, Y.; Watari, J. Biotransformation of hop-derived monoterpene alcohols by lager yeast and their contribution to the flavor of hopped beer. *J Agric Food Chem*. **2010**, 58, 5050-5058. https://doi.org/10.1021/jf1000524.
200. Li, L.; Cheng, P.; You, X.; Fan, Q.; Zhu, A.; Zhang, J. Research Progress in Functional Active Ingredients in Jiangxiang Baijiu. *Liquor-Making Science & Technology*. **2023**, 09, 109-113. https://doi.org/10.13746/j.njkj.2022289.
201. Hong, J.; Zhao, D.; Sun, B. Research Progress on the Profile of Trace Components in Baijiu. *Food Reviews International*. **2023**, 39, 1666-1693. https://doi.org/10.1080/87559129.2021.1936001.
202. Xu, Z.; Chen, Y.; Zhou, Z.; Tang, Q. Research of Functional Ingredients in Notable Chinese Liquor-JIANNANCHUN Liquor. *Sichuan Food and Fermentation*. **2008**, 44, 24-27.
203. Fu, H.; Chen, X.; Zhao, Y.; Chen, M.; Zhou, D.; Jia, W. Research progress on the component analysis in Baijiu. *Food and Fermentation Industries*. **2021**, 47(21), 320-327. https://doi.org/10.13995/j.cnki.11-1802/ts.027379.
204. Du, P.; Jiao, G.; Zhang, Z.; Wang, J.; Li, P.; Dong, J.; Wang, R. Relationship between Representative Trace Components and Health Functions of Chinese Baijiu: A Review. *Fermentation*. **2023**, 9, 658. https://doi.org/10.3390/fermentation9070658.
205. Zhao, D.; Shi, D.; Sun, J.; Li, H.; Zhao, M.; Sun, B. Quantification and cytoprotection by vanillin, 4-methylguaiacol and 4-ethylguaiacol against AAPH-induced abnormal oxidative stress in HepG2 cells. *RSC Advances*. **2018**, 8, 35474-35484. https://doi.org/10.1039/C8RA06505E.
206. Zhao, D.; Sun, J.; Sun, B.; Zhao, M.; Zheng, F.; Huang, M.; Sun, X.; Li, H. Intracellular antioxidant effect of vanillin, 4-methylguaiacol and 4-ethylguaiacol: three components in Chinese Baijiu. *RSC Advances*. **2017**, 7, 46395-46405. https://doi.org/10.1039/C7RA09302K.
207. Fujitaka, K.; Otani, H.; Jo, F.; Jo, H.; Nomura, E.; Iwasaki, M.; Nishikawa, M.; Iwasaka, T.; Das, D.K. Modified resveratrol Longevinex improves endothelial function in adults with metabolic syndrome receiving standard treatment. *Nutrition Research*. **2011**, 31, 842-847. https://doi.org/10.1016/j.nutres.2011.09.028.
208. Rauf, A.; Imran, M.; Abu-Izneid, T.; Iahtisham Ul, H.; Patel, S.; Pan, X.; Naz, S.; Sanches Silva, A.; Saeed, F.; Rasul Suleria, H.A. Proanthocyanidins: A comprehensive review. *Biomedicine & Pharmacotherapy*. **2019**, 116, 108999. https://doi.org/10.1016/j.biopha.2019.108999.
209. Sharma, K.; Kumar, V.; Kaur, J.; Tanwar, B.; Goyal, A.; Sharma, R.; Gat, Y.; Kumar, A. Health effects, sources, utilization and safety of tannins: a critical review. *Toxin Reviews*. **2019**, 40, 432-444. https://doi.org/10.1080/15569543.2019.1662813.

210. Zhang, M.; Chen, Z.; Tian, D.; Pan, L.; Liu, W. Research Progress on Healthy Functional Components of Nongxiangxing Baijiu. *China Food Safety Magazine*. **2022**, 15, 110-113. https://doi.org/10.16043/j.cnki.cfs.2022.15.031
211. Zhu, Z.; Rao, J.; Zhang, J.; Li, D.; Wang, J.; Cai, F.; Zhang, R.; Xu, J. Research progress of health factors in Huangjiu. *China Brewing*. **2021**, 40(05), 26-31. https://doi.org/10.11882/j.issn.0254-5071.2021.05.005.
212. Shi, X. 2022. Study on the analysis and detection ofdifferent quality Baijiu and the method ofremoving harmful substances. thesis, Xiangtan University,Hunan, China.
213. Vahdat-Lasemi, F.; Aghaee-Bakhtiari, S.H.; Tasbandi, A.; Jaafari, M.R.; Sahebkar, A. Targeting interleukin-beta by plant-derived natural products: Implications for the treatment of atherosclerotic cardiovascular disease. *Phytother Res*. **2021**, 35, 5596-5622. https://doi.org/10.1002/ptr.7194.
214. Wu, X.; Zhou, X.; Chen, C.; Mao, W. Systematic Investigation of Quercetin for Treating Cardiovascular Disease Based on Network Pharmacology. Combinatorial *Chemistry & High Throughput Screening*. **2019**, 22, 411-420. https://doi.org/10.2174/1386207322666190717124507.
215. Jiang, C.; Sun, T.; Xiang, D.; Wei, S.; Li, W. Anticancer Activity and Mechanism of Xanthohumol: A Prenylated Flavonoid From Hops (Humulus lupulus L.). *Front. Pharmacol*. **2018**, 9, 530. https://doi.org/10.3389/fphar.2018.00530.
216. Samuels, J.S.; Shashidharamurthy, R.; Rayalam, S. Novel anti-obesity effects of beer hops compound xanthohumol: role of AMPK signaling pathway. *Nutrition & Metabolism*. **2018**, 15, 42. https://doi.org/10.1186/s12986-018-0277-8.
217. Liou, S.; Nguyen, T.; Hsu, J.; Sulistyowati, E.; Huang, S.; Wu, B.; Lin, M.; Yeh, J. The Preventive Effects of Xanthohumol on Vascular Calcification Induced by Vitamin D3 Plus Nicotine. *Antioxidants*. **2020**, 9, 956. https://doi.org/10.3390/antiox9100956.
218. Sun, X.; Shen, D.; Shi, T.; Cui, G. Research on decomposition of triglycerides activities and α-glycosidase inhibitory of sulfide and pyrazine compositions in sesame-flavor liquor. *Liquor Making*. **2104**, 41, 56-59.
219. Gao, C.; Tian, T.; Xin, Y. The activities of the extracts of zhimaxiang Baijiu (sesame-flavor liquor) and the 4 kinds of characteristic compounds. *Liquor-Making Science & Technology*. **2015**, 250, 61-64. https://doi.org/10.13746/j.njkj.2015016.
220. Hu, Z.; Chen, F.; Guo, X.; Zhao, T.; Luo, Z.; Wu, D. Research progress on formation mechanism of health factor 2,3,5,6-tetramethylpyrazine in Baijiu. *China Brewing*. **2023**, 43(01), 27-33. https://doi.org/10.11882/j.issn.0254-5071.2024.01.005.
221. Zhang, Q.; Zhu, T.; Huang, M.; Wei, J.; Wu, J.; Huo, J. Intracellular Antioxidant Activity of Two Terpenoids in Baijiu. *Food Science & Nutrition*. **2020**, 41(09), 66-73. https://doi.org/10.7506/spkx1002-6630-20190412-176.
222. Henriques, J.F.; Serra, D.; Dinis, T.C.P.; Almeida, L.M. The Anti-Neuroinflammatory Role of Anthocyanins and Their Metabolites for the Prevention and Treatment of Brain Disorders. *International Journal of Molecular Sciences*. **2020**, 21, 8653. https://doi.org/10.3390/ijms21228653.
223. Huang, W.; Liu, Y.; Wang, J.; Wang, X.; Li, C. Anti-Inflammatory Effect of the Blueberry Anthocyanins Malvidin-3-Glucoside and Malvidin-3-Galactoside in Endothelial Cells. *Molecules*. **2014**, 19, 12827-12841. https://doi.org/10.3390/molecules190812827.
224. Pu, J.; Wang, M.; Chen, J.; Wang, M. Simultaneous detection of amide herbicides and their intermediates in beerby solid-phase extraction coupled with gas chromatography-massspectrometry. *Journal of Food Safety & Quality*. **2018**, 9(06), 1369-1376. https://doi.org/10.7666/d.D01436670.
225. Lv, H.; Geng, D.; Xie, H.; Guo, C.; Cai, Y.; Yang, G. Optimization of detection method and migration amount for phthalate acid esters in beer. *China Brewing*. **2021**, 40(08), 157-162. https://doi.org/10.11882/j.issn.0254-5071.2021.08.028.
